# Future projection of climate extremes across contiguous northeast India and Bangladesh

**DOI:** 10.1038/s41598-023-42360-2

**Published:** 2023-09-20

**Authors:** Ashesh Rudra Paul, Rajib Maity

**Affiliations:** Department of Civil Engineering, Indian Institute of Technology Kharagpur, Kharagpur, West Bengal 721302 India

**Keywords:** Hydrology, Climate change

## Abstract

In recent times, India has experienced a significant increase in the frequency and intensity of extreme weather events, particularly in northeast India (NEI), an area known for its rich natural resources. Despite the geographic continuity of NEI and Bangladesh, previous studies have failed to consider their interconnectedness, resulting in an incomplete understanding of the situation. To bridge this gap, a comprehensive study encompassed the entire NEI, including West Bengal and Bangladesh (hereafter referred to as NEIB). This study examined climate extremes in NEIB, utilizing 12 temperature-based and 8 precipitation-based indices developed by the Expert Team on Climate Change Detection and Indices. Analysis was performed on temperature and precipitation data obtained from the India Meteorological Department and Bangladesh Meteorological Department covering the period 1981–2021. Additionally, climate projections from 14 Global Climate Models participating in the CMIP6 were incorporated for the period 2015–2100, considering four different Shared Socioeconomic Pathways (SSPs) scenarios. Findings revealed that under the SSP585 scenario, a substantial rise of 4 °C in maximum temperatures and 5.5 °C in minimum temperatures by the end of the twenty-first century. Warming indices, such as the summer days index, indicated an expected increase of 53 days, while the Warm spell days index was estimated to rise by approximately 2 days. Heavy precipitation days (R20mm) were projected to increase by up to 14 days, with a notable impact in Meghalaya. While the number of rainy days is expected to decrease, the overall magnitude of precipitation is anticipated to remain relatively stable. Notably, the Simple daily intensity index demonstrated a rise of 2.4 mm/day compared to the current baseline of 14.4 mm/day. These projected changes have significant ramifications for water resources, agriculture, health, and infrastructure in the region.

## Introduction

As a consequence of climate change, there is a growing concern that extreme weather and climate events may increase in frequency, duration, spatial extent, and intensity^1,2^. These extreme events, with their multifaceted adverse impacts on both society and the environment, pose intricate challenges for resource management, particularly in developing nations like India^3^. India, being highly vulnerable to extreme weather and climate events, experiences significant consequences, especially in regions dependent on natural resources. One such region is Northeast India (NEI), known for its abundant greenery, which is susceptible to climate variability and extreme events due to its unique geographic location^4–9^. The NEI region encompasses eight states, covering a total geographical area of 26.2 million hectares with a population of approximately 45 million based on the 2011 census.

The NEI region is characterized by the presence of two major rivers, namely the Brahmaputra and Barak, which play a vital role in the local system and significantly contribute to the livelihoods of the inhabitants. The local population heavily relies on the region's natural resources for their sustenance and income, rendering them particularly vulnerable to the impacts of climate change and extreme weather events. In addition to experiencing diverse climate regimes, the NEI region is significantly influenced by the southwest monsoon, which occurs from June to September and substantially impacts its climatic conditions. More than 60% of cultivated land in NEI is dependent on rain-fed agriculture, making it highly susceptible to climate variability and the potential impacts of climate change^10^.

There have been several noteworthy observable climate change incidents in NEI^11^. Very recently, in June 2022, the NEI region experienced record-breaking rainfall, leading to severe flooding, particularly in Assam (URL: https://www.eastmojo.com/northeast-news/2022/06/17/__trashed-41/, accessed in January 2023). Despite stable long-term precipitation trends, regions, including neighboring Bangladesh, are witnessing declining rainfall coupled with rising temperatures^12–16^. According to the India Meteorological Department (IMD) reports, Assam experienced a monsoon with a 22% rainfall deficit and a 21% to 30% deficit in the six other northeastern states. Alongside the rise in the frequency of extreme precipitation events, there has also been a reported increase in the intensity of extreme temperature events over time^3,17,18^. These incidents underscore the urgent need for a comprehensive assessment of the impacts of climate change, as extreme weather events are reported to be increasing drastically in many regions of NEI^6,12,19^.

Recent decades have witnessed an upward trend in maximum and minimum temperatures (T_max_ and T_min_) in the NEI region^20–23^, with statistically significant increases observed since the 1980s^24–27^. Current global climate projections indicate that escalating temperatures will result in a higher occurrence of extreme events like heavy rainfall, floods, forest fires, tropical storms, and prolonged droughts^28–31^. Rising temperatures contribute to increased evapotranspiration rates, altered rainfall patterns, and more frequent and extended droughts and floods^32–36^. This alarming trend in rising temperatures is not limited to NEI alone; the neighboring country of Bangladesh also faces significant vulnerability to climate change and global warming. Bangladesh is also known to be highly susceptible to the impacts of climate change and global warming, primarily because of its unique geographic location, extensive floodplains, low elevation, high poverty levels, and heavy reliance on natural resources and services^37,38^. Bangladesh, being a neighboring country of India, especially with NEI, shares significant similarities in their climatic conditions due to their close proximity. However, this geographical location and climate make Bangladesh one of the most susceptible countries to natural disasters like sea-level rise, cyclones, and floods^39^.

Therefore, the incidents mentioned above are indicative of climate change for the entire NEIB region, emphasizing the need for heightened awareness and action. To effectively manage these extreme events and develop effective long-term adaptation plans, it is crucial to have accurate information about the frequency and severity of climate extremes at the regional and local scales. The Expert Team on Climate Change Detection and Indices (ETCCDI) has developed 27 core climate change indices, encompassing 11 for precipitation and 16 for temperature measurement. These indices provide a framework for defining and assessing changes in the Earth's climate system (URL: http://etccdi.pacificclimate.org/list_27_indices.shtml). These statistically reliable indices are designed to capture crucial characteristics such as the frequency, intensity, and duration of daily temperature and precipitation extremes^26,40–44^. However, research focusing on daily precipitation and temperature in the NEI region has been limited, with only a few studies conducted in the past decade. Indeed, the existing studies on the spatiotemporal variability of precipitation and temperature using ETCCDI indices have primarily focused on specific regions or states within NEI^18,40,45–49^. While these studies have provided valuable insights into local climate dynamics, there remains a significant gap in comprehensive research that encompasses the entire NEI and Bangladesh region. Understanding the impact of climate change on this entire geographic region is crucial. For instance, several studies^39,47,50,51^ have explored the spatiotemporal variability of precipitation and temperature extremes using ETCCDI indices. Still, they have generally focused on specific areas or stations. For example, Shrivastava^52^ discovered distinct changes in precipitation extremes over Shillong and Imphal through the use of two weather stations. Their findings showed heightened intensity, duration, and frequency of precipitation, coupled with reduced total precipitation and more cumulative dry days. Similarly, Basher et al.^12^ employed only eight stations to identify variations in extremes in the northeastern region of Bangladesh.

However, it is essential to note that when it comes to assessing climatic changes and their characteristics, the majority of studies exclude Bangladesh or NEI rather than considering them as a geographically continuous land mass. While the aforementioned studies provide accurate information about the specific areas surrounding the stations they use, they may not fully represent the entire geographic region. Therefore, it is vital to recognize the interconnectivity and interdependence of these two regions (NEI and Bangladesh). This study aims to overcome existing limitations by conducting a comprehensive assessment of climate change indices related to precipitation and temperature across the entire NEI and Bangladesh region.

A novel research study is introduced, treating NEI and Bangladesh as an integrated study area due to their geographical and climatic interconnectedness. While prior research has largely focused on climate projections within distinct geographical zones, our study acknowledges the inherent links between NEI and Bangladesh, necessitating a unified methodology. Our distinctive investigation aims to bridge this gap by offering a comprehensive understanding of how climate influences the broader region. By dissecting the intertwined factors shaping NEI and Bangladesh, we contribute to an enriched comprehension of the complex climatic dynamics within this shared geographical expanse. The study’s overall objective is to perform a comprehensive assessment of climate change indices pertaining to precipitation and temperature across the entire NEIB region, achieved through investigating observed datasets and simulations from a multi-model ensemble (MME) of GCMs. Within this context, the study pursues two specific objectives:

1. Assess the future trends and changes in a comprehensive set of 12 selected extreme temperature and 8 extreme precipitation indices within the NEIB region.
2. Investigate the association between the chosen extreme temperature and precipitation indices during both the present and future periods.

**Figure 1:**
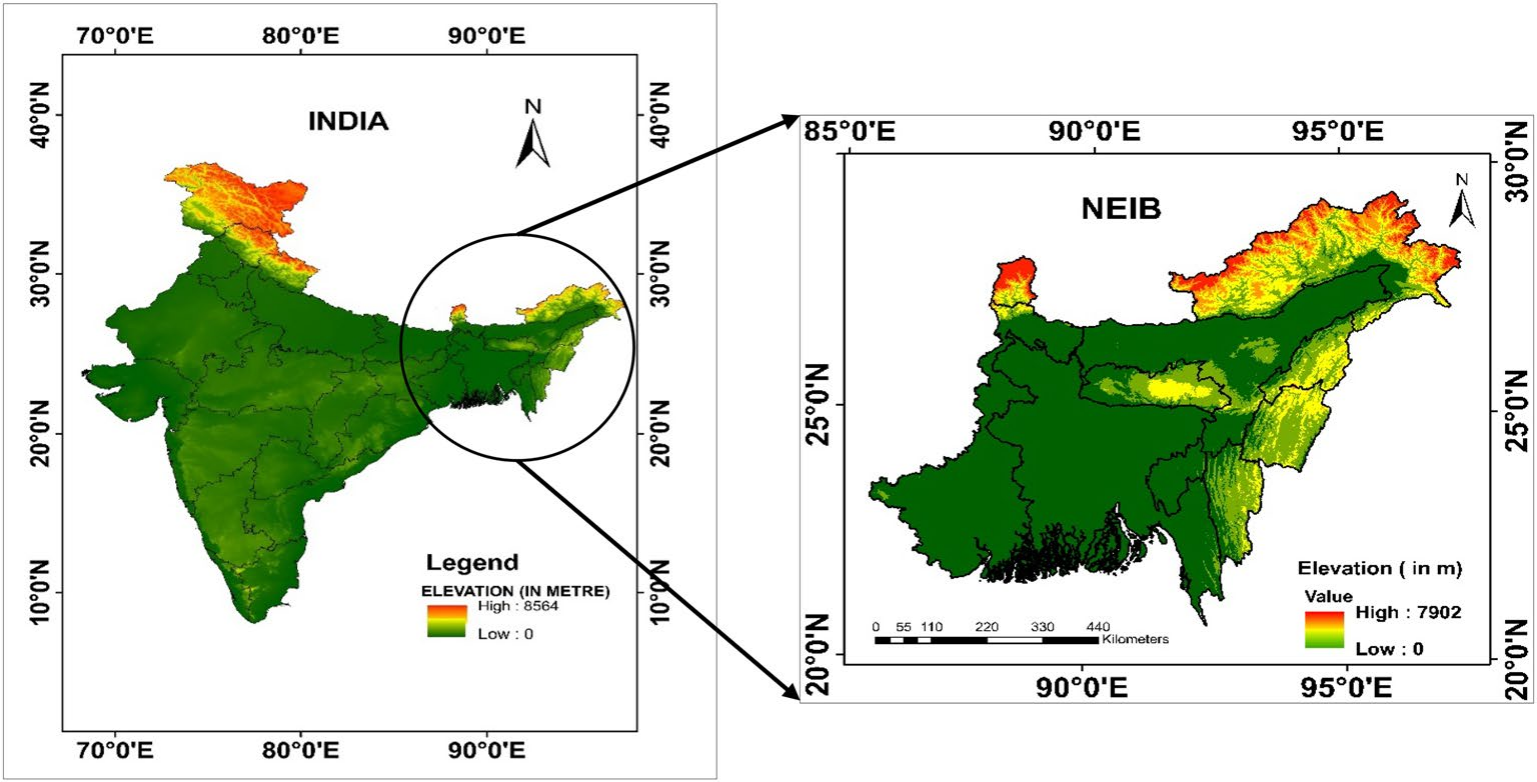
Location map of the study area (Northeast India, West Bengal, Bangladesh). *Source* SRTM DEM with 30 m Resolution.

By pursuing this goal, our research addresses current research gaps and provides a holistic understanding of how climate intricately shapes the interconnected NEI and Bangladesh regions (Fig. 1).

## Results

**Figure 2:**
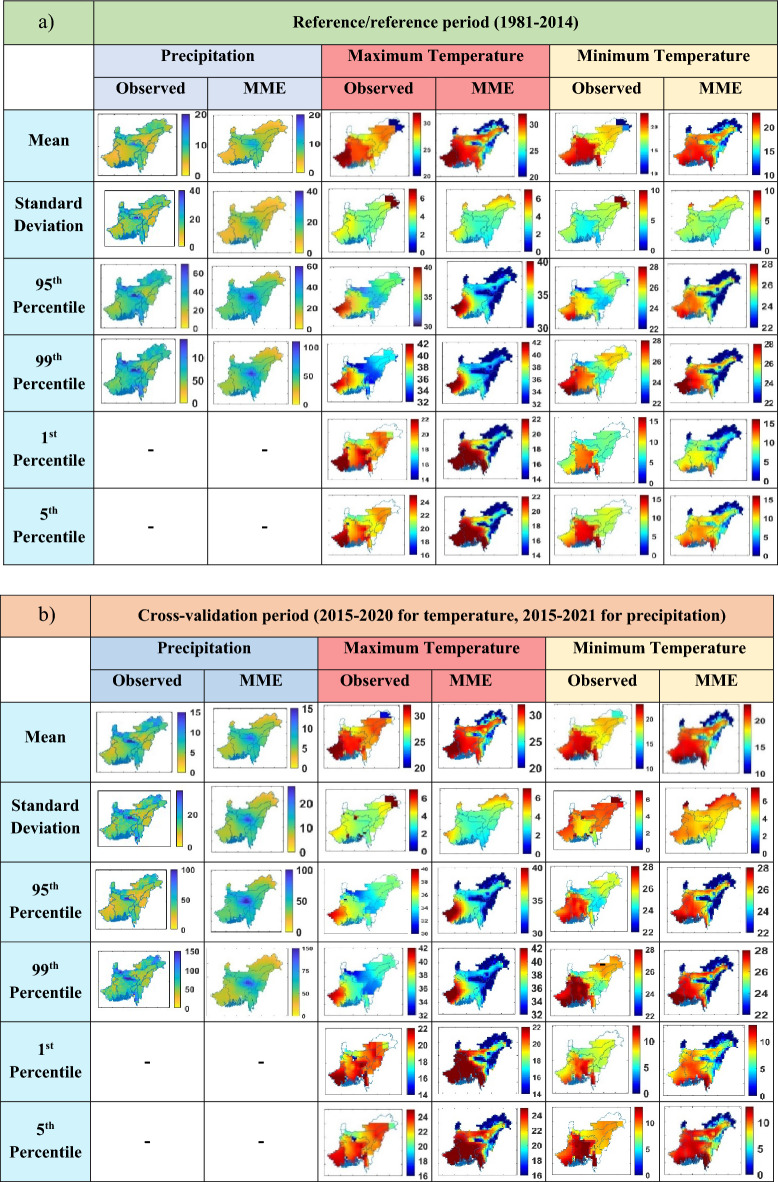
Statistical comparison between observed data and MME of the 14 GCM models simulation over NEIB during the (**a**) reference period (1981–2014) and (**b**) cross-validation period, when GCM simulations are considered as future period. The figure indicates a satisfactory association between the MME of the 14 GCM models and observed datasets of precipitation and temperature.

The performance of the GCMs in simulating current climate conditions is assessed in order to determine reliability for future projections. Figure 2 represents a spatial comparison between the observed data and MME of the 14 GCM models, focusing on precipitation, maximum temperature, and minimum temperature. The comparison is shown in Fig. 2 using a two-time span; one is the 1981–2014 timescale, which is used as the reference period, and another is 2015–2021 (2015–2020 for temperature), which is the cross-validation period. The comparison between the GCM-simulated and observed precipitation and temperature data is shown by various statistical parameters, such as (a) mean, (b) standard deviation, (c) 95th Percentile, (d) 99th Percentile, (e) 1st Percentile, (f) 5th Percentile. The result indicates a satisfactory association between the GCM simulations and the observed data for both precipitation and temperature, suggesting that the models adequately capture the variability of these variables. Overall, the evaluation of the GCMs’ performance in reproducing current climate conditions provides confidence in their ability to project future climate scenarios.

### Spatially regional average of temperature and precipitation indices

**Figure 3:**
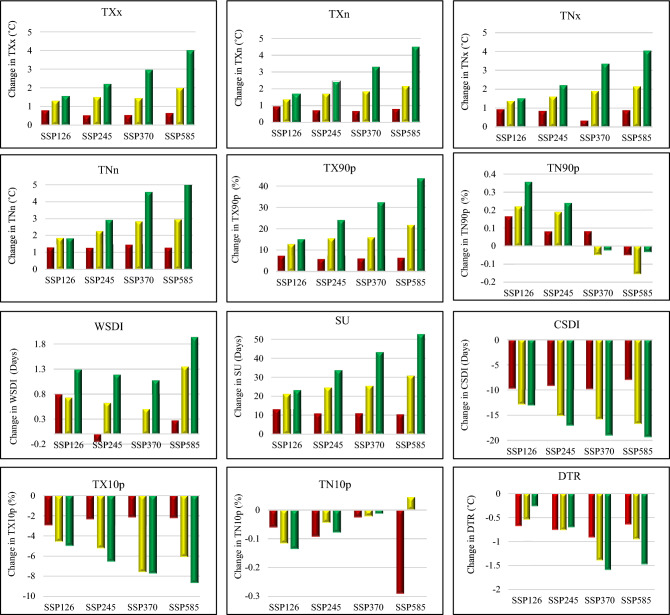
Under four different scenarios, the average magnitude of the various temperature indices changed throughout three different Epochs. Epochs 1, 2, and 3 are represented by the red, yellow, and green bars, respectively. The change of SU, WSDI, and CSDI is given in Days; the change of TX90p, TX10p, TN90p, and TN10p is given in %; the change of TXx, TXn, TNx, TNn, DTR is given in °C.

The spatially regional average of temperature indices exhibit both increasing and decreasing tendencies in the future compared to the reference period, as depicted in Fig. 3. For the NEIB region, the maximum changes are predominantly observed in Epoch 3 for most cases. The impact of climate change becomes evident as the mean annual temperature in the study area changes more significantly in scenario SSP585 than in scenario SSP126. Under the SSP126 scenario, the maximum positive changes in temperature indices are observed in Epoch 3. Specifically, TXx increases by 1.57 °C, TXn by 1.67 °C, TNx by 1.52 °C, and TNn by 1.83 °C, compared to the reference period. Conversely, under the SSP585 scenario, the maximum positive changes were higher, with TXx increasing by 4.02 °C, TXn by 4.51 °C, TNx by 4.05 °C, and TNn by 5.5 °C in Epoch 3. While both maximum and minimum temperatures increase in the future, the diurnal temperature range (DTR) shows a decreasing in the future compared to the reference period. In SSP126, the maximum negative change is observed in DTR (− 0.27 °C), and in the SSP585, the maximum negative change in observed in DTR (− 1.5 °C).

The increase in warm indices (TX90p, TN90p, WSDI, and SU) and the decrease in cold indices (TX10p, TN10p, and CSDI) can be attributed to the overall increase in maximum and minimum temperatures in the study area. Under the SSP126 scenario, the maximum positive changes were observed for TX90p (15.15%), TN90p (0.36%), WSDI (1.29 days), and SU (23.28 days) in Epoch 3. However, negative changes are observed for TX10p (− 5%), TN10p (− 0.14%), and CSDI (− 13.08 days). In comparison, under the SSP585 scenario, even more significant positive changes are observed for TX90p (43.68%), WSDI (1.942 days), SU (52.92 days), and TN10p (0.001%). Conversely, negative changes were observed for TN90p (− 0.034%), TX10p (-8.7%), and CSDI (− 19.39 days) in Epoch 3.

**Figure 4:**
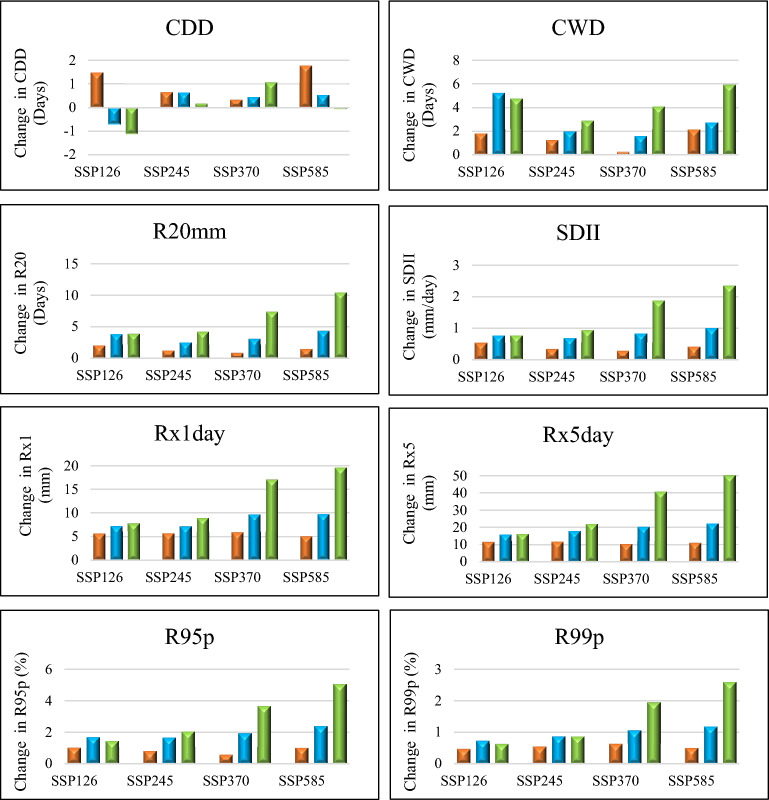
Under four different scenarios, the average magnitude of the various precipitation indices changed throughout three different Epochs. Epochs 1, 2, and 3 are represented by the orange, blue, and green bars, respectively. The change of CWD, CDD, and R20mm is given in Days; the change of SDII is given in mm/day; the change of Rx1day, Rx5day is given in mm; the change of R95p and R99p is given in %.

When comparing the precipitation indices to the temperature indices, it is generally observed that the spatially average precipitation indices exhibit positive changes, as illustrated in Fig. 4. However, the index for consecutive dry days (CDD) shows a decrease in its average value in the future for most scenarios, except for scenario SSP370 where the CDD value increases compared to the reference period. The maximum difference in CDD value among the four scenarios is − 1.12 days, 0.17 days, 1.07 days, and − 0.08 days for Epoch 3, respectively. Regarding scenario SSP126, the maximum positive changes in mean value are observed in the indices for CWD (5.24 days), R20 mm (3.93 days), SDII (0.77 mm/day), Rx1 day (7.84 mm), Rx5 day (16.26 mm), R95p (1.71%), and R99 (0.74%) for Epoch 3 (except for CWD, R95p, and R99p which were found in Epoch 2). On the other hand, under scenario SSP585, even maximum positive changes are observed in the indices for CWD (5.92 days), R20 mm (10.45 days), SDII (2.36 mm/day), Rx1 day (19.60 mm), Rx5 day (50.27 mm), R95p (5.07%), and R99 (2.6%) are observed for Epoch 3. These changes were generally higher compared to the other SSP scenarios.

The findings highlight that under scenario SSP585, the magnitude of changes in precipitation indices surpasses that of temperature indices compared to other scenarios. Specifically, while the CDD index shows a negative change, indicating a decrease in the number of dry periods, the other precipitation indices demonstrate positive changes. This suggests a notable increase in the occurrence of extreme precipitation events within the study area. These results underscore the potential for more intense rainfall and an increased frequency of heavy precipitation events in the future. This highlights the critical need to comprehend and adapt to these changes effectively in order to mitigate their potential impacts.

### Long-term variations in temperature indices

The long-term analysis of temperature indices in the NEIB under different Epochs and scenarios reveals a statistically significant rising trend in TXx, TXn, TNx, and TNn across a major part of the study area (Figs. S1b–S4b). The maximum temperature change is found mainly in the north of Arunachal Pradesh, Meghalaya, north Sikkim, and some southern parts of West Bengal (WB) (Fig. S1a). The maximum positive change in TXx is found at approximately 1.9 °C, 3 °C, 4 °C, and 5 °C for four different scenarios during Epoch 3. TXx exhibits a significant rising trend, with a maximum increase of + 0.065 °C per year during Epoch 3 (Fig. S1b). Under scenario SSP585, TXn demonstrates the maximum positive change of 5.5 °C compared to all other Epochs (Fig. S2). Similarly, TXn exhibits a significant rising trend of up to 0.06 °C per year under SSP585 during Epoch 3 (Fig. S2b). TNx shows a maximum positive change of 5.2 °C under SSP585 during Epoch 3 (Fig. S3a), accompanied by a significant rising trend of up to 0.05 °C per year (Fig. S3b). In the case of TNn, the maximum positive change is 7.2 °C under SSP585 during Epoch 3, primarily observed in north Sikkim and north Arunachal Pradesh (Fig. S4a). Significant rising trends of up to 0.08 °C per year are observed in Bangladesh, WB, some parts of Mizoram, and Tripura under SSP585 during Epoch 3 for TNn (Fig. S4b).

**Figure 5:**
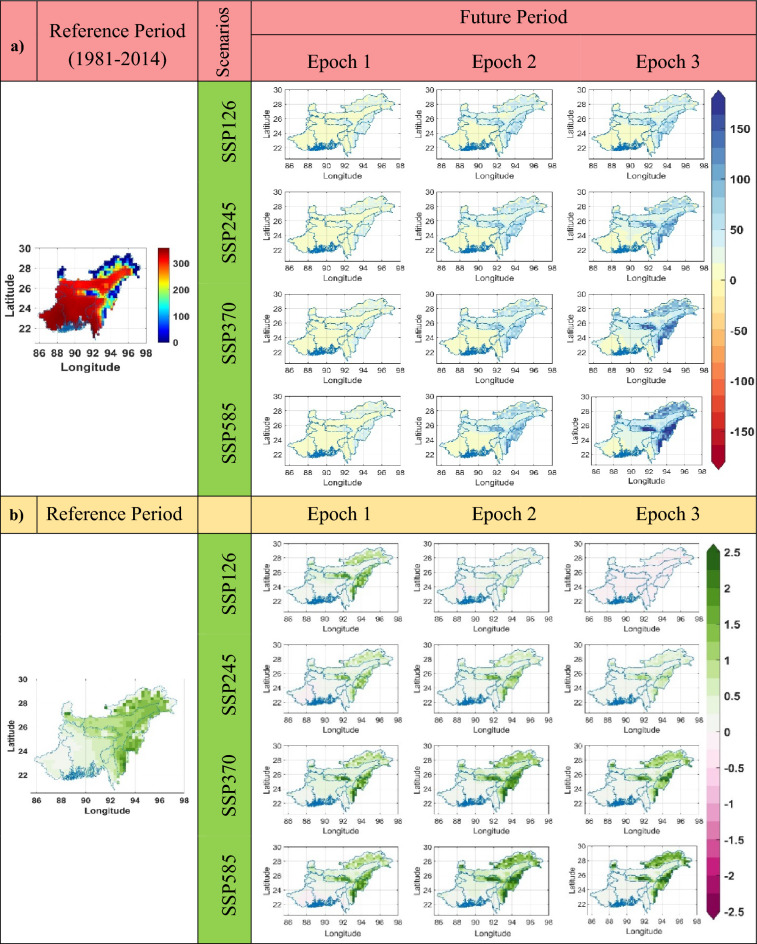
(**a**) Change of the SU (in Days) during three different future Epochs as compared to the reference period, derived from the MME of the 14 GCM models for four SSP scenarios (CMIP6). (**b**) Trend (in Days/year) of SU during the reference period and three future Epochs. The green, purple, and white colors represent a statistically significant increasing, decreasing trend, and insignificant trend, respectively. The maximum change of SU in the future is increased (up to 190 Days), with a positive trend of up to 3.11 days/year for Epoch 3 under scenario SSP585.

The Diurnal temperature range (DTR) indices in the NEIB exhibit both increasing and decreasing trends across all scenarios (Fig. 5b). However, in terms of magnitude, the future DTR values generally indicate a decreasing tendency compared to the reference period (Fig. 5a). Notably, regions such as Bangladesh, Meghalaya, and Nagaland show the most prominent positive trends in DTR. Conversely, negative trends are observed in areas including Arunachal Pradesh, Sikkim, portions of southern West Bengal, and Assam. The DTR index demonstrates a significant rising trend of up to 0.06 °C per year in certain regions, indicating an increasing temperature difference between day and night. Conversely, a significant declining trend of up to − 0.04 °C per year is observed in other areas (Fig. 5b).

The rising trends in maximum temperature (TX) and minimum temperature (TN) directly influenced summer days (SU) in the NEIB region (Fig. 5). The SU value exhibits a significant increase in regions such as Sikkim, Meghalaya, Arunachal Pradesh, Nagaland, Mizoram, and Manipur, indicating a higher number of days classified as summer days (Fig. 5a). However, the rate of increase in SU days is relatively lower in Bangladesh, Tripura, and West Bengal (WB) compared to other states in the NEIB region. The maximum positive changes in SU days for the four scenarios are up to 80 days, 120 days, 160 days, and 190 days, respectively, observed during Epoch 3. This indicates a substantial increase in the number of summer days compared to the reference period. Moreover, SU shows a significant rising trend of 1 day/year, 1.1 days/year, 2.1 days/year, and 3.1 days/year for the SSP126, SSP245, SSP370, and SSP585 scenarios, respectively (Fig. 5b). These trends highlight the increasing frequency and duration of summer days in the NEIB region, emphasizing the potential impacts of rising temperatures on the local climate.

In addition, an increase in Warm Spell Duration Index (WSDI) and a reduction in Cold Spell Duration Index (CSDI) coincide with the rise in TX and TN across the NEIB region (Figs. S6a, S7a). In the northern regions of Bangladesh, Tripura, Meghalaya, Assam, Manipur, Mizoram, and Nagaland, the most notable positive change in the WSDI is observed to be approximately 3.5 days under the SSP585 scenario during Epoch 3. Conversely, West Bengal, Arunachal Pradesh, and Sikkim show the most negative change (up to − 1.1 days) (Fig. S6a). In Bangladesh and the northern portion of WB, a significant rising trend is observed, with an increase of up to 0.8 days/year (Fig. S6b). On the other hand, under the SSP585 scenario, the CSDI exhibits a significant decrease, indicating a reduction in the duration of cold spells. The maximum declining trend is observed up to − 0.3 days per year, as depicted in Fig. S12b. In specific regions such as Bangladesh, Tripura, Meghalaya, Nagaland, and certain parts of Assam, the most noteworthy negative changes in CSDI are observed. These changes amount to approximately − 18 days, − 19 days, − 21 days, and − 22 days for the four scenarios, respectively, as illustrated in Fig. S7a. These findings highlight the increasing occurrence of warm spells and the diminishing occurrence of cold spells in the study area, indicating a shift towards warmer conditions.

Similar to CSDI, WSDI, and SU, the increase in maximum temperature (TX) and minimum temperature (TN) is also accompanied by changes in the frequency of warm and cold days and nights. The number of warm days (TX90p) exhibits a significant positive change, with maximum increases of 20%, 30%, 40%, and 50% for the four scenarios, respectively, across the entire NEI region, including the major part of Bangladesh, under Epoch 3 (Fig. S8a). However, the change in TX90p is relatively less in West Bengal and some parts of Bangladesh. In specific regions such as Arunachal Pradesh, Sikkim, Nagaland, and a major portion of Assam, TX90p shows a significant declining trend, with a maximum decrease of up to − 0.25%/year detected. Conversely, a significant rising trend of up to 0.9%/year is detected in a major portion of the study area (Fig. S8b). The current study also detected that the cold days (TX10p) in the future are decreasing compared to the reference period. This change is mainly found in the major portion of Arunachal Pradesh and some parts of Assam and Nagaland. The maximum negative change is detected as − 6%, − 8.1%, − 9.2%, and 9.6% for the respective four scenarios (Fig. S10a). Furthermore, a significant positive trend up to 0.12%/year is detected, and on the other hand, a significant negative trend up to -0.05%/year is seen for TX10p (Fig. S10b).

Regarding TN90p, the analysis reveals a maximum positive change (up to 0.62%) is observed in regions including Bangladesh, Assam, Mizoram, Manipur, and Nagaland, as shown in Fig. S9a. Conversely, a significant negative trend (up to − 0.4% per year) in TN90p is detected across a major part of the study area, particularly in Tripura, Nagaland, Mizoram, and Manipur. On the other hand, a significant positive trend (up to 0.1% per year) is observed in Meghalaya, northern West Bengal, the upper part of Bangladesh, and the lower part of Assam, as depicted in Fig. S9b.

For TN10p, the analysis reveals that the maximum negative change compared to the reference period is observed up to − 0.2% in northern West Bengal, the upper part of Bangladesh, Sikkim, Arunachal Pradesh, Assam, and Nagaland for all scenarios, as shown in Fig. S11a. Notably, there is a significant positive trend of up to 0.15% per year (under SSP126) and a negative trend of up to − 0.35% per year (under SSP585) for TN10p, as illustrated in Fig. S11b.

### Long-term variations in precipitation indices

The study investigated precipitation extremes over a long-term period (1981–2100) and revealed that most of the study area did not show a significant change. However, the number of consecutive dry days (CDD) exhibited both positive and negative changes across different regions, depending on the scenarios considered (Fig. S12). The highest positive change (up to 2 days) in CDD was observed in Meghalaya, Nagaland, Tripura, and Manipur. In comparison, the maximum negative change (up to − 3.5 days) was detected in Sikkim, West Bengal, Bangladesh, and certain parts of Assam and Arunachal Pradesh (see Fig. S12a). Furthermore, a notable positive trend (up to 0.15 days/year) was detected in North West Bengal and the northernmost part of Bangladesh, while a significant negative trend (up to − 0.2 days/year) was observed in a substantial portion of the study area, particularly in Arunachal Pradesh and Assam, during Epoch 3 under the SSP126 scenario. For the other three scenarios, the maximum negative trend ranged from − 0.2 days/year to − 0.3 days/year for Epoch 3 (see Fig. S12b).

The analysis of future CWD indicates a positive change compared to the reference period across various scenarios. The maximum positive change (up to 15 days) is observed in South WB, along with certain parts of Bangladesh and Mizoram (see Fig. S13a). Conversely, a negative change (up to 2 days) is detected predominantly in the Assam and Arunachal Pradesh regions of the NEI. Notably, only specific areas within the region exhibit a significant trend in CWD. A significant positive trend (up to 0.4 days/year) is observed in Sikkim, Manipur, Bangladesh, and certain parts of Assam and Arunachal Pradesh (Fig. S13b).

The regions of Meghalaya exhibit the most notable changes in Rx5day and RX1day (refer to Figs. S14 and S15). Specifically, certain parts of Meghalaya demonstrate a maximum positive change (up to 20 mm, 16 mm, 40 mm, and 42 mm) in Rx1 day across the four different scenarios for Epoch 3 (Fig. S15a). Under the SSP585 scenario, a significant positive trend (up to 0.6 mm/day) is observed in Sikkim, Meghalaya, and Assam. In comparison, the remaining areas of the study exhibit a declining trend (up to − 0.3 mm/day) (Fig. S15b). Similarly, Rx5 day displays a comparable trend to Rx1 day in the Meghalaya region, but the magnitude of change is more significant in Rx5 day. The maximum positive change for different scenarios in Rx5 day is 40 mm, 43 mm, 100 mm, and 120 mm, respectively, for Epoch 3 (Fig. S14a). Furthermore, significant positive trends up to 1.6 mm/ year and significant negative trends up to − 1.2 mm/day are detected under various scenarios (Fig. S14b).

The study area shows an increase in the number of very wet days (R95p) and extreme wet days (R99p) in certain regions, such as Sikkim, Assam, Meghalaya, Tripura, north WB, and some parts of Arunachal Pradesh (Figs. S16, S17). Across the four scenarios for Epoch 3, the maximum positive change (up to 2.5%, 4%, 5.2%, and 8%) in R95p is detected (Fig. S16a). Significant positive trends (up to 0.085%/year) in R95p are identified in certain parts of WB, Bangladesh, and Assam. In contrast, a significant declining trend (up to − 0.06%/year) is observed in Manipur, Mizoram, and certain parts of Bangladesh (Fig. S16b). The upper part of the study area, including Sikkim, Assam, North West Bengal, Nagaland, and Arunachal Pradesh, exhibits the maximum positive change in R99p, ranging from 1.1 to 5% for Epoch 3 under the four scenarios (Fig. S17a). A significant rising trend (up to 0.065%/year) is detected in West Bengal. In contrast, Tripura, Mizoram, and certain parts of Bangladesh and WB show a significant negative trend (up to − 0.04%/year) in R99p (Fig. S17b).

The study area demonstrates a significant increase in the number of days with precipitation greater than 20 mm (R20mm) in Bangladesh, Tripura, and Mizoram under different scenarios (refer to Fig. S18). The maximum positive change is up to 7, 8, 10, and 14 days for four scenarios in Sikkim, Bangladesh, Tripura, Assam, Mizoram, and Manipur (Fig. S18a). Furthermore, R20mm exhibits a significant positive trend (up to 0.3 days/year) in Tripura, Assam, and Sikkim for all scenarios. Conversely, negative trends (up to − 0.1 days/year) are detected in certain parts of Bangladesh and Assam (Fig. S18b).

**Figure 6:**
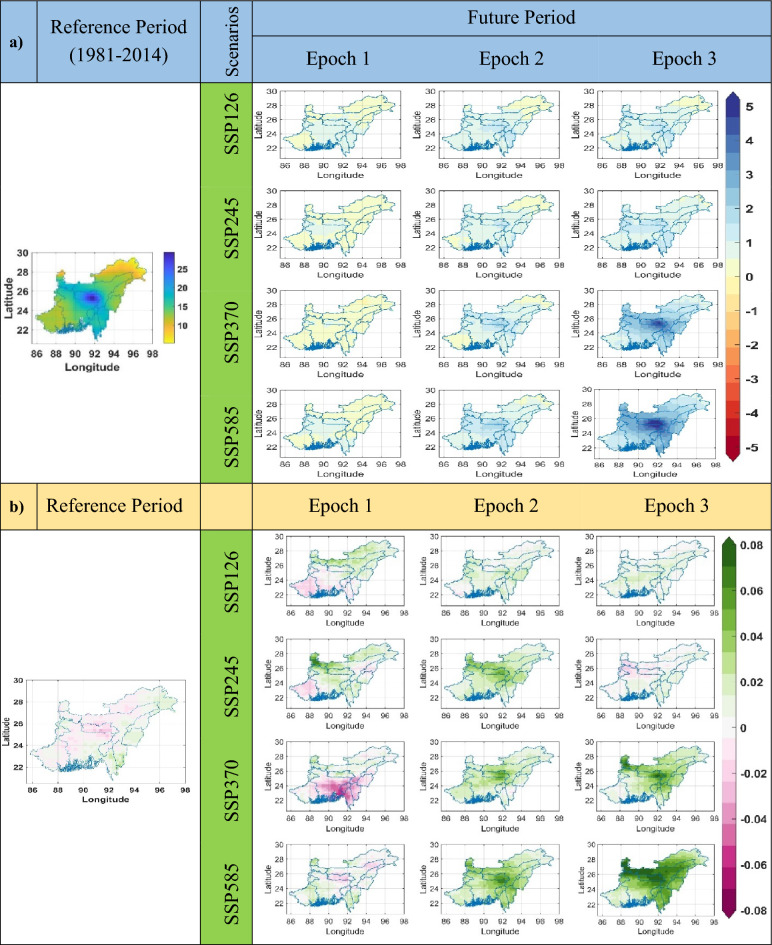
(**a**) Change of the SDII (in mm/day) in three different future Epochs as compared to the reference period, derived from the MME of the 14 GCM models for four SSP scenarios (CMIP6). (**b**) Trend (in mm/day/year) of SDII during the reference period and three different future Epochs. The green, purple, and white colors represent a statistically significant increasing, decreasing trend, and insignificant trend, respectively. The maximum change of SDII in the future is found to be increased (up to 5.2 mm/day) with a positive trend of up to 0.08 mm/day/year for Epoch 3 under scenario SSP585.

The upper part of the study area, including Sikkim and North West Bengal, showcases the maximum positive change in the Simple Daily Intensity Index (SDII) (refer to Fig. 6). Across the four scenarios, the maximum positive changes are 1.4 mm/day, 1.6 mm/day, 4.2 mm/day, and 5.2 mm/day, respectively, for Epoch 3 (Fig. 6a). SDII exhibits a significant positive trend (up to 0.08 mm/day/year) in Meghalaya. In contrast, a significant negative trend (up to − 0.01% mm/day/year) is observed in Sikkim, Arunachal Pradesh, and Nagaland under different scenarios (Fig. 6b).

### Correlation between extreme precipitation and temperature indices

Supplementary Table T1 presents the association between extreme precipitation and temperature indices during reference and future periods, considering four distinct SSP scenarios. The strength of the association is indicated by the Pearson correlation coefficient, while the significance is represented by the p-value, considering a 5% significance level. The extreme precipitation and temperature indices were found to be more strongly correlated in the future scenarios SSP370 and SSP585, with significant negative correlations observed between warm and cold extremes. Specifically, hot days were positively correlated with hot extremes and negatively correlated with cool extremes. Moreover, extreme precipitation indices demonstrate a positive correlation with hot extreme temperature indices and a negative correlation with cool extreme temperature indices.

In SSP126, a highly significant negative correlation of − 0.54 was observed between R20 and DTR, and a highly significant positive correlation of 0.37 existed between R20 and WSDI. In SSP245, a strong positive correlation of 0.48 existed between R20 and the extreme precipitation index CDD. Within SSP370, TN90p exhibited a significant positive correlation of 0.73 with R99p and a significant negative correlation of − 0.64 with CSDI. R99p also displayed a highly significant positive correlation of 0.74 with TX90p and R99p. In SSP585, a highly significant positive correlation of 0.74 was observed between R99p and TNx, while a highly significant negative correlation of − 0.58 existed between R20 and DTR. However, for most instances of CWD and CDD, the correlation was insignificant for future and reference periods.

## Discussions

Noteworthy shifts in climate extremes have been detected in the NEIB through the utilization of gridded climate datasets. The current analysis provides good visualizations (maps) of future magnitude change with respect to the reference period, along with trends in both temperature and precipitation extremes. These visualizations enable the comparison of variations occurring in various regions within the study area. The majority of the temperature indices in the area under consideration show clearly rising tendencies. The research results are consistent with the increasing trends in TX, and TN in the NEIB, especially in the NEI part. In the NEIB region, there is an apparent increase in the number of warm nights (TN90P), warm days (TX90P), summer days index (SU), and warm spell days index (WSDI). Conversely, declining trends can be seen in TN10P, TX10P, and CSDI. Our results are consistent with the overall trends of these indices at the regional level, suggesting a decreasing trend for cold indices and an increasing trend for warm indices.

In general, the observed changes in temperature extremes are consistent with what is expected, given the increase in global mean temperature. In addition, several studies found significant upward trends in mean temperature over the past 50–100 years^28,53,54^ as well as minimum and maximum temperatures. Fewer precipitation indicators showed significant changes than temperature indices, which did so over most of the region. The increase in SDII, R95P, R99P, and R20mm and a decrease in CDD indicate that the climatically humid region will become even wetter in the warmer future. This is particularly concerning given the region's climatic humidity and the complex river systems in the hilly terrain of Northeast India (NEI).

The result also shows that with compare to the whole study area, the NEI part is more vulnerable to the impacts of climate change compared to the West Bengal and Bangladesh part due to its unique geographic and ecological characteristics. NEI is characterized by its hilly terrain, complex river systems, and high levels of biodiversity, which make the region more susceptible to the effects of climate change. Additionally, the landscape of NEI is a perfect example of how orographic effects contribute to the region’s uneven distribution of precipitation, causing one side of the hills to receive maximum precipitation while the other side is in a rain shadow. Because of this, there is also a difference in the temperature of the region. Moreover, NEI is situated in a region that is projected to experience more remarkable climate change impacts due to its proximity to the Himalayas, which act as a barrier to the monsoon winds. This results in variations in precipitation patterns, with some areas experiencing prolonged periods of drought while others face frequent flooding. These factors make the NEI part more vulnerable to climate change compared to the relatively low-lying regions of West Bengal and most of Bangladesh. However, this does not mean that West Bengal and Bangladesh are not affected by climate change. They are also experiencing changes in temperature and precipitation patterns, which can have significant consequences for agriculture, water resources, and public health.

The implications of these climate extremes on the NEIB region are far-reaching. Agriculture, being a vital sector in the region, is particularly vulnerable. It is expected that changes and unpredictability of extremes such as rainfall and temperature variability will impact crop development and, ultimately, the region's food security. The NEIB region heavily relies on agriculture for its economy and food supply. However, alternations in climatic conditions and the occurrence of extreme weather events such as heavy rainfall, droughts, and heat waves can significantly impact agricultural productivity, crop growth, and food security. For example, prolonged dry spells can lead to crop failures and water scarcity, while heavy rainfall and floods can damage crops and infrastructure. The changes and unpredictability in rainfall and temperature patterns have made it difficult for farmers to plan and manage their crops effectively, leading to lower yields and increased risk of food shortages.

Therefore, it is crucial to understand the potential impacts of climate change on agriculture in the NEIB region and develop adaptation strategies to mitigate these impacts. Moreover, the increase in warm days and nights can have significant ramifications for various industries, including energy, water supply, and transportation. The change in precipitation and temperature has both direct and indirect effects on human health also. Due to the above illustration and other implications of shifting extremes, there is a need to improve the current forecasting system and raise awareness at all levels, particularly regional, local, and across many sectors, to manage the risks. Effective risk management strategies, adaptation measures, and the integration of climate change considerations into development planning are essential for mitigating the impacts and building resilience in the NEIB region. Collaborative efforts between India and Bangladesh are also vital in addressing the shared challenges posed by climate change in the region.

## Concluding remarks

India’s Northeast is unique in the perspectives of climate change impact analysis. In most studies, this region is considered a part of the entire Indian mainland but always remains in the shadow of findings/discussions for other regions. Geographically, consideration of India’s Northeast along with Bangladesh is essential to study the change in climatic conditions, particularly its spatial variation. This is due to the unique geographical features of the region—barrier by the Himalayas in the north and east, bounded by the Bay of Bengal in the south, and one of the very specific regions to first welcome the monsoon season in the subcontinent. World’s highest rainfall-receiving region (Cherrapunji, approximate annual precipitation = 11,777 mm) also lies in this region. Denoting this entire region as NEIB, this study considers a total of 20 temperature and precipitation based climate change indices to analyze their annual and long-term variability across NEIB. It was required to stitch the data from two different sources. The holistic analysis, as presented in this study, helps to understand the crucial changes and the pattern of their evolution in climate change through the aforementioned 20 climate change indices. The following are the key findings of our investigation:

1. Across nearly all of NEIB, substantial changes have been observed in all temperature-based climate change indices by the end of the twenty-first century. The majority of indices demonstrate an upward trend, with maximum and minimum temperatures increasing to 4.0 °C and 5.5 °C, respectively, in the future (Epoch 3) under scenario SSP585. It is anticipated that the minimum temperature will experience a higher rate of increase compared to the maximum temperature, leading to a decrease in the Diurnal temperature range (DTR) of up to 1.5 °C.
2. Warming indices such as the Summer days index (SU) and Warm spell day index (WSDI) are projected to increase in the future for Epoch 3 under scenario SSP585. In the future, the number of SU will increase by 53 days compared to the current value of 260 days across the NEIB region. Additionally, the spatially averaged value of WSDI increased by approximately 2 days, whereas it is currently 17 days. The NEI regions of Sikkim, Meghalaya, Arunachal Pradesh, Nagaland, and Manipur are expected to be the most affected by temperature changes in the future under the SSP585 scenario.
3. Climate trends in NEIB reveal an apparent rise in annual precipitation at the end of the twenty-first century. However, it is also observed that the number of precipitation days is expected to decrease in the future. This shift is accompanied by an increase in the intensity of rainfall events, particularly in Meghalaya state. This implies that despite a smaller number of precipitation days, when rainfall does occur, it is more intense and concentrated, highlighting the heightened risk of heavy rainfall events in the region. Under the SSP585 scenario, the spatially averaged value of the Simple daily intensity index (SDII) rises by 2.4 mm/day compared to its current value of 14.4 mm/day.
4. In Meghalaya state, specifically, the current value of the SDII is approximately 27 mm/day, and under the SSP585 scenario, it is projected to increase by 5.2 mm/day in the future. This substantial increase in SDII indicates a warm invitation to potential flooding events in Meghalaya and neighboring states such as Assam, Tripura, and Mizoram.
5. The analysis of extreme precipitation and temperature indices across different SSP scenarios revealed significant correlations. The future scenarios SSP370 and SSP585 demonstrated stronger correlations, with negative associations between warm and cold extremes. Notable correlation values include a highly significant negative correlation between R20 and DTR (− 0.58) in SSP585 and a significant positive correlation between R99p and TNx (0.74) in SSP370. These correlations suggest potential impacts on the frequency of heavy precipitation events and the intensity of maximum temperatures in the respective scenarios.

In conclusion, the MME projection approach has been employed to evaluate the implications of climate change in the NEIB region. The results indicate that the region is exceptionally susceptible to climate change, with notable projections of shifts in temperature, precipitation, and extreme weather events. These changes are likely to have significant impacts on water resources, agriculture, health, and infrastructure. Overall, the outcomes of this study highlight the urgent need for action to address the impacts of climate change in NEIB. Through the adoption of appropriate adaptation strategies and prompt action, the region can mitigate risks and build a more sustainable future for its habitants.

## Data and methods

The unique relationship between Bangladesh and India region is characterized by their extensive shared land border, the fifth longest globally, spanning over 4096 km. Out of this, 1879 km forming the boundary between NEI and Bangladesh^55^. This geographical proximity also leads to shared climatic conditions, making a joint study imperative for accurate climate change representation. Therefore, any comprehensive investigation into the region's climate change scenario must encompass both NEI and Bangladesh. Besides NEI, Bangladesh shares a significant border with West Bengal, a state in eastern India that shares similar geographical and climatic traits. The study primarily focuses on the NEIB region from 1981 to 2100 (as shown in Fig. 1). NEI's subtropical climate is influenced by the Himalayas to the north and the Meghalaya Plateau to the south. In contrast, West Bengal experiences tropical weather with varied topography, and Bangladesh's low-lying plains are nourished by Himalayan rivers^56^.

The NEIB region, spanning from 21° 50′ N to 29° 34′ N and 85° 34′ E to 97° 50′ E, witnesses a humid subtropical climate with high humidity, moderate temperatures, and distinct seasonal rainfall patterns. Monsoon rains dominate the summer months of June through September, accounting for over 75% of annual rainfall. Summer temperatures range from 20 to 35 °C in NEI and West Bengal, while Bangladesh sees averages from 7.2 to 31.1 °C during winter and summer, respectively.

Precipitation patterns vary across the regions. NEI experiences no less than 1000 mm annually, with Cherrapunji recording 11,465 mm due to its location on the Shillong Plateau^57,58^. Guwahati receives 1717 mm due to its rain shadow location^5,57,59^. Bangladesh sees diverse rainfall patterns, with the western region receiving around 1400 mm and the eastern region experiencing over 4400 mm, owing to the Meghalaya Plateau’s influence^60^.

### Data

To investigate temperature and precipitation changes over the NEIB, this study utilizes 14 CMIP6-GCM datasets (listed in Table 1) and gridded observed datasets based on rain gauge observations.

**Table 1:** Lists the reporting institutions, nations, spatial and temporal for each CMIP6 GCM with its variant label.

Sl. no	Name of the GCM	Modeling center/Nation	Data resolution		Variant label
			Spatial	Temporal	
1	ACCESS-ESM1-5	Commonwealth Scientific and Industrial Research Organization/Australia	0.25° × 0.25°	DAILY	r1i1p1f1
2	BCC-CSM2-MR	Beijing Climate Center China Meteorological Administration/China	0.25° × 0.25°	DAILY	r1i1p1f1
3	CanESM5	Canadian Centre for Climate Modelling and Analysis/Canada	0.25° × 0.25°	DAILY	r1i1p1f1
4	CMCC-ESM2	Centro Euro-Mediterraneo sui Cambiamenti Climatici, Lecce 73,100, Italy	0.25° × 0.25°	DAILY	r1i1p1f1
5	EC-Earth3-Veg-LR	EC–EARTH consortium/Europe	0.25° × 0.25°	DAILY	r1i1p1f1
6	FGOALS-g3	Chinese Academy of Sciences/China	0.25° × 0.25°	DAILY	r3i1p1f1
7	GFDL-ESM4	NOAA Geophysical Fluid Dynamics Laboratory/USA	0.25° × 0.25°	DAILY	r1i1p1f1
8	INM-CM4-8	Institute for Numerical Mathematics, Russian Academy of Science/Russia	0.25° × 0.25°	DAILY	r1i1p1f1
9	IPSL-CM6A-LR	L’Institute Pierre–Simon Laplace/France	0.25° × 0.25°	DAILY	r1i1p1f1
10	KACE-1–0-G	National Institute of Meteorological Sciences (NIMS) and Korea Meteorological Administration (KMA)/Korea	0.25° × 0.25°	DAILY	r1i1p1f1
11	MIROC6	Japan Agency for Marine-Earth Science and Technology, Atmosphere and Ocean Research Institute, The University of Tokyo/Japan	0.25° × 0.25°	DAILY	r1i1p1f1
12	MPI-ESM1-2-LR	Max Planck Institute for Meteorology/Germany	0.25° × 0.25°	DAILY	r1i1p1f1
13	NorESM2-MM	Norwegian Climate Centre/Norway	0.25° × 0.25°	DAILY	r1i1p1f1
14	UKESM1_0_LL	Met Office Hadley Centre/UK	0.25° × 0.25°	DAILY	r1i1p1f2

#### Observation data

The study utilized observational grid datasets of daily gridded precipitation for NEI and WB obtained from the India Meteorological Department (IMD) (URL: https://www.imdpune.gov.in/Clim_Pred_LRF_New/Grided_Data_Download.html, accessed in January 2023). These datasets had a spatial resolution of 0.25° × 0.25° and covered the period 1981–2021. The maximum and minimum temperature data for NEI and WB were also obtained from IMD from 1981 to 2020, with a spatial resolution of 1° × 1°. Similarly, the observed precipitation and temperature data for Bangladesh were acquired from the Bangladesh Meteorological Department (BMD) (URL: https://datalibrary.bmd.gov.bd/SOURCES/.Bangladesh/.BMD/.daily/.rainfall/.rfe_merged/datafiles.html, accessed in January 2023). These datasets had a spatial resolution of 0.05° × 0.05° and covered the same period. The existing 1° × 1° and 0.05° × 0.05° grid data were regrided to 0.25° × 0.25° using the linear interpolation method to maintain uniform spatial resolution across all meteorological datasets.

#### Model data

This study utilizes a total of 14 CMIP6-GCMs to assess daily precipitation, maximum and minimum temperatures in the NEIB region (URL: https://www.nccs.nasa.gov/services/data-collections/land-based-products/nex-gddp-cmip6, accessed in January 2023). The data for these climate simulations were obtained from the NASA Earth Exchange Global Daily Downscaled Projections (NEX-GDDP)-CMIP6 dataset, which is a global downscaled climate scenario dataset generated as part of the CMIP6 initiative to support the Sixth Assessment Report of the Intergovernmental Panel on Climate Change (IPCC AR6)^61^. The NEX-GDDP-CMIP6 dataset incorporates GCM runs performed by the participating models, and these runs were specifically bias-corrected using the ‘Bias-Correction Spatial Disaggregation’ (BCSD) technique^62–64^. The study employs a daily spatial resolution that covers the period from 1981 to 2100 and a temporal resolution of 0.25° × 0.25°. Table 1 in the study presents the names of the CMIP6 models, the respective institutions, the nations associated with each model, and the horizontal resolutions of each model. It should be noted that the ensemble sizes of the CMIP6 models vary, and for consistency, this study accounts for the r1i1p1f1 variance of the first element of all models. In some cases, due to data availability, the variance r2i1p1f1 and r3i1p1f1 are only used for two models. To examine the precipitation and temperature projections, this study considers four Shared Socioeconomic Pathway (SSP) scenarios: SSP126 (low forcing scenario), SSP245 (moderate forcing scenario), SSP370 (high forcing scenario), and SSP585 (very high forcing scenario). These scenarios provide different assumptions about future socioeconomic development and greenhouse gas emissions, allowing for a comprehensive analysis of the potential climate outcomes projected by the 14 CMIP6 GCMs.

### Methodology

#### Extreme climate change indices

In this study, climate projections are derived from the Multi-Model Ensemble (MME) mean values obtained from simulations conducted by various GCMs participating in the CMIP6. The MME mean values provide an aggregated representation of the climate projections generated by multiple models, enhancing the robustness of the analysis. To ensure consistency and comparability, a reference period of 1981–2014 is utilized to analyze future changes in extreme temperature and precipitation indices. The entire specified time frame from 2015 to 2100 is extrapolated to estimate these changes. The study uses the baseline from 1981 to 2014 to estimate future changes in various extreme temperature and precipitation indices. The future time span is divided into three consecutive epochs: Epoch 1: 2015–2040; Epoch 2: 2041–2070; and Epoch 3: 2071–2100. These epochs provide distinct time periods within the future timeframe, allowing for a more detailed examination of climate changes over time. Regarding the selection of the cross-validation period, the years 2015–2021 (2015–2020 for temperature) are chosen as a reference period relative to the present time, 2023. However, it is important to note that the CMIP6 datasets compile the reference experiment for each model from 1950 to 2014. Therefore, for the purpose of cross-validation in this study, precipitation data from 2015 to 2021 and temperature data from 2015 to 2020 are utilized. By adhering to these methodologies and timeframes, the study ensures consistency and accuracy in analyzing future climate projections and comparing them to the reference period, enabling a robust assessment of changes in extreme temperature and precipitation indices.

Within the specified region, climate change indices for the period of 1981–2100 are computed using annual precipitation and temperature data at each grid point. These indices are based on the established guidelines by the Expert Team on Climate Change Detection and Indices (ETCCDI). They are designed to provide statistically reliable metrics for assessing climate change. A total of 12 temperature-based indices and 8 precipitation-based indices are utilized in the analysis. These indices, as outlined in Tables 2 and 3, respectively, capture various important characteristics of temperature and precipitation events. They encompass a wide range of factors, such as the magnitude of temperature extremes, the severity of heat waves and cold spells, the duration of extreme events, and the frequency of both extreme and moderate temperature and precipitation events. These indices provide valuable insights into the nature and extent of climate change, allowing for a more comprehensive understanding of the potential impacts and risks associated with future climate scenarios. The use of these indices is supported by previous research and their established relevance in climate science studies^18,41,46,65–68^.

**Table 2:** Selected extreme temperature indices, along with their ETCCDI definitions.

Indicator name	Indices	ETCCDI definitions	Units
Annual maximum of daily maximum temperature	TXx	The highest temperature of the Annual daily maximum temperature	°C
Annual minimum of daily maximum temperature	TXn	The lowest temperature of the Annual daily maximum temperature	°C
Annual maximum of daily minimum temperature	TNx	The highest temperature of the Annual daily minimum temperature	°C
Annual minimum of daily minimum temperature	TNn	The lowest temperature of the Annual daily minimum temperature	°C
Diurnal Temperature Range	DTR	Difference between the maximum temperature and the minimum temperature	°C
Number of warm days	TX90p	Percentage of days where the daily maximum temperature is greater than the 90th Percentile of the base period	%
Number of days with warm nights	TN90p	Percentage of days where the daily minimum temperature is greater than the 90th Percentile of the base period	%
Warm spell days index	WSDI	At least six consecutive days with a daily maximum temperature greater than the 90th Percentile of the base period	Days
Summer days index	SU	Days with an annual maximum temperature greater than 25°C	Days
Cold spell days index	CSDI	At least six consecutive days with a daily minimum temperature lower than the 10th Percentile of the base period	Days
Number of cold days	TX10p	Percentage of days where the daily maximum temperature is lower than the 10th Percentile of the base period	%
Number of days with cold nights	TN10p	Percentage of days where the daily minimum temperature is lower than the 10th Percentile of the base period	%

**Table 3:** Selected extreme precipitation indices, along with their ETCCDI definitions.

Indicator name	Indices	Definitions	Units
Max 1 day precipitation amount	RX1 day	Monthly maximum 1 day precipitation	mm
Max 5 days precipitation amount	RX5 day	Monthly maximum 5 days precipitation	mm
Very wet days	R95p	Percentage of wet days exceeding the 95th Percentile of the base period	%
Extreme wet days	R99p	Percentage of wet days exceeding the 99th Percentile of the base period	%
Simple daily intensity index	SDII	Annual total precipitation divided by the number of wet days	mm/day
Consecutive dry days	CDD	Maximum number of consecutive days with precipitation below 1 mm	Days
Consecutive wet days	CWD	Maximum number of consecutive days with precipitation above 1 mm	Days
Number of heavy precipitation days	R20mm	Annual count of days when precipitation is greater than 20 mm	Days

### Trend analysis of different indices

The study focused on conducting trend analysis of various climate change indices across the NEIB region. It has specifically investigated 20 precipitation and temperature-based climate change indices using the MME of 14 GCMs from the sixth phase of the Coupled Model Intercomparison Project (CMIP6). These indices included maximum and minimum temperature, heat waves, cold spells, and extreme precipitation events. To assess the significance of trends in the time series data, the study employed the Mann–Kendall (MK) test. The MK test is a widely used non-parametric statistical test in climate science, specifically used to determine the presence of trends in time series datasets. Additionally, Sen’s slope, another non-parametric method, was used to estimate the magnitude of the trend in the analyzed dataset. Together with these two methods, the study aimed to evaluate and quantify the trends and changes in climate variables within the NEIB region, providing valuable insights into the potential impacts of climate change in the area. The study also computed gridded values of the extreme indices with spatial and temporal precision for the study area, which allowed for a detailed investigation of the spatial distribution of trends.

Furthermore, the study incorporated the Pearson correlation coefficient to evaluate the correlation between extreme precipitation and temperature indices in the NEIB region. By determining the statistical significance of the correlation at a 5% significance level, the analysis shed light on the potential associations between extreme events, both in the reference period and the future. By employing these methods, the study provided a comprehensive analysis of climate change indices, enabling a deeper understanding of the changing climate dynamics in the NEIB region and the potential implications for extreme precipitation and temperature events.

### Supplementary Information

Supplementary Information.

## Data Availability

The data supporting the findings of this research paper include gridded daily precipitation and maximum/minimum temperature datasets for the Northeast Indian (NEI) and West Bengal (WB) regions, which were obtained from the India Meteorological Department (IMD). These datasets are accessible at the URL: https://www.imdpune.gov.in/Clim_Pred_LRF_New/Grided_Data_Download.html. Additionally, the research incorporates observation data for Bangladesh, consisting of gridded daily precipitation and temperature datasets, acquired from the Bangladesh Meteorological Department (BMD). The data files for Bangladesh can be accessed at the URL: https://datalibrary.bmd.gov.bd/SOURCES/.Bangladesh/.BMD/.daily/.rainfall/.rfe_merged/datafiles.html. Furthermore, the study utilizes model data in the form of climate simulations from 14 CMIP6-GCMs, obtained from the NASA Earth Exchange Global Daily Downscaled Projections (NEX-GDDP)-CMIP6 dataset. These climate simulations provide valuable insights for the study and are available at the URL: https://www.nccs.nasa.gov/services/data-collections/land-based-products/nex-gddp-cmip6.

## Code availability

The code used for this study is available upon request from the corresponding author.
