# Supplementary material for: Future projection of climate extremes across contiguous northeast India and Bangladesh
Source: Sci Rep. 2023 Sep 20;13:15616. doi: 10.1038/s41598-023-42360-2 (PMC10511735; doi:10.1038/s41598-023-42360-2)
Supplement: Supplementary file 1 — Supplementary Information. [file 41598_2023_42360_MOESM1_ESM.docx]

**Supplementary Document**

**Future Projection of Climate Extremes across Contiguous Northeast India and Bangladesh**

Ashesh Rudra Paul and Rajib Maity^[[1]](#footnote-1)^

Department of Civil Engineering, Indian Institute of Technology

Kharagpur, Kharagpur-721302, West Bengal, India

This file contains supplementary Tables 1 and Supplementary Figures from 1-18, as mentioned in the main article. The list of Figures and table in order of their presentation are as follows:

**List of Tables:**

**Table T1:** Matrix of Pearson's correlation coefficients between the extreme climate indices for the reference and future period under four different scenarios with a 5% significance level. (The green shaded indicates the significance at a 5% significance level).

**List of Figures:**

**Figure S1**: **(a)** Change of the TXx (in °C) during three different future Epochs as compared to the reference period, derived from the MME of the 14 GCM models for four SSP scenarios (CMIP6). **(b)** Trend (in °C/year) of TXx, during the reference period and three different future Epochs. The green, purple, and white color represent a statistically significant increasing, decreasing trend, and insignificant trend, respectively. The maximum change of TXx in the future is increased (up to 5°C), with a positive trend of up to 0.065°C/year for Epoch 3 under scenario SSP585.

**Figure S2**: **(a)** Change of the TXn (in °C) during three different future Epochs as compared to the reference period, derived from the MME of the 14 GCM models for four SSP scenarios (CMIP6). **(b)** Trend (in °C/year) of TXn, during the reference period and three future Epochs. The green, purple, and white color represent a statistically significant increasing, decreasing trend, and insignificant trend, respectively. The maximum change of TXn in the future is increased (up to 5.5°C), with a positive trend of up to 0.06°C/year for Epoch 3 under scenario SSP585.

**Figure S3**: **(a)** Change of the TNx (in °C) during three different future Epochs as compared to the reference period, derived from the MME of the 14 GCM models for four SSP scenarios (CMIP6). **(b)** Trend (in °C/year) of TNx during the reference period and three different future Epochs. The green, purple, and white color represent a statistically significant increasing, decreasing trend, and insignificant trend, respectively. The maximum change of TNx in the future is increased (up to 5.2°C), with a positive trend of up to 0.05°C/year for Epoch 3 under scenario SSP585.

**Figure S4:** **(a)** Change of the TNn (in °C) during three different future Epochs as compared to the reference period, derived from the MME of the 14 GCM models for four SSP scenarios (CMIP6). **(b)** Trend (in °C/year) of TNn during the reference period and three different future Epochs. The green, purple, and white color represent a statistically significant increasing, decreasing trend, and insignificant trend, respectively. The maximum change of TNn in the future is increased (up to 7.2°C), with a positive trend of up to 0.08°C/year for Epoch 3 under scenario SSP585.

**Figure S5:** **(a)** Change of the DTR (in ˚C) during three different future Epochs as compared to the reference period, derived from the MME of the 14 GCM models for four SSP scenarios (CMIP6). **(b)** Trend (in ˚C/year) of DTR during the reference period and three different future Epochs. The green, purple, and white color represent a statistically significant increasing, decreasing trend, and insignificant trend, respectively. The maximum change of DTR in the future is decreased (up to -1.2 ˚C), mainly with a negative trend of up to -0.04 ˚C /year for Epoch 3 under scenario SSP585.

**Figure S6:** **(a)** Change of the WSDI (in Days) during three different future Epochs as compared to the reference period, derived from the MME of the 14 GCM models for four SSP scenarios (CMIP6). **(b)** Trend (in Days/year) of WSDI during the reference period and three future Epochs. The green, purple, and white color represent a statistically significant increasing, decreasing trend, and insignificant trend, respectively. The maximum change of WSDI in the future is increased (up to 3.5 Days), with a positive trend of up to 0.8 days/year for Epoch 3 under scenario SSP585.

**Figure S7: (a)** Change of the CSDI (in Days) during three different future Epochs as compared to the reference period, derived from the MME of the 14 GCM models for four SSP scenarios (CMIP6). **(b)** Trend (in Days/year) of CSDI during the reference period and three future Epochs. The green, purple, and white colors represent a statistically significant increasing, decreasing, and insignificant trend, respectively. The maximum change of WSDI in the future is decreased (up to -22 Days), with a negative trend of up to -0.018 days/year for Epoch 3 under scenario SSP585.

**Figure S8:** **(a)** Change of the TX90p (in %) during three different future Epochs as compared to the reference period, derived from the MME of the 14 GCM models for four SSP scenarios (CMIP6). **(b)** Trend (in %/year) of TX90p during the reference period and three future Epochs. The green, purple, and white color represent a statistically significant increasing, decreasing trend and insignificant. The maximum change of TX90p in the future is increased (up to 50 %), with a positive trend of up to 0.9 %/year for Epoch 3 under scenario SSP585.

**Figure S9:** **(a)** Change of the TN90p (in %) during three different future Epochs as compared to the reference period, derived from the MME of the 14 GCM models for four SSP scenarios (CMIP6). **(b)** Trend (in %/year) of TN90p during the reference period and three future Epochs. The green, purple, and white color represent a statistically significant increasing, decreasing trend, and insignificant trend, respectively. The maximum change of TN90p in the future is decreased (up to -0.15 %), with a positive trend of up to 0.62 %/year for Epoch 3 under scenario SSP585.

**Figure S10: (a)** Change of the TX10p (in %) during three different future Epochs as compared to the reference period, derived from the MME of the 14 GCM models for four SSP scenarios (CMIP6). **(b)** Trend (in %/year) of TX10p during the reference period and three future Epochs. The green, purple, and white color represent a statistically significant increasing, decreasing trend, and insignificant trend, respectively. The maximum change of TX10p in the future is decreased (up to -9.6 %), with a decreasing trend of up to -0.05 %/year for Epoch 3 under scenario SSP585.

**Figure S11:** **(a)** Change of the TN10p (in %) during three different future Epochs as compared to the reference period, derived from the MME of the 14 GCM models for four SSP scenarios (CMIP6). **(b)** Trend (in %/year) of TN10p during the reference period and three future Epochs. The green, purple, and white color represent a statistically significant increasing, decreasing trend, and insignificant trend, respectively. The maximum change of TN10p in the future is both increasing (up to 0.065%) and decreasing (up to -0.07 %) in nature, with a negative trend of up to -0.35 %/year for Epoch 3 under scenario SSP585.

**Figure S12:** **(a)** Change of the CDD (in Days) during three different future Epochs as compared to the reference period, derived from the MME of the 14 GCM models for four SSP scenarios (CMIP6). **(b)** Trend (in Days/year) of CDD during the reference period and three future Epochs. The green, purple, and white color represent a statistically significant increasing, decreasing trend, and insignificant trend, respectively. The maximum change of CDD in the future is increased (up to 2 days) and decreasing (up to -3.5 days) in nature, with a mixed trend for Epoch3 under scenario SSP585.

**Figure S13:** **(a)** Change of the CWD (in Days) during three different future Epochs as compared to the reference period, derived from the MME of the 14 GCM models for four SSP scenarios (CMIP6). **(b)** Trend (in Days/year) of CWD during the reference period and three different future Epochs. The green, purple, and white color represent a statistically significant increasing, decreasing trend, and insignificant trend, respectively. The maximum change of CWD in the future is increased (up to 15 days), with a positive trend of up to 0.3 days/year for Epoch 3 under scenario SSP585.

**Figure S14:** **(a)** Change of the Rx5day (in mm) during three different future Epochs as compared to the reference period, derived from the MME of the 14 GCM models for four SSP scenarios (CMIP6). **(b)** Trend (in mm/year) of Rx5day during the reference period and three future Epochs. The green, purple, and white color represent a statistically significant increasing, decreasing trend, and insignificant trend, respectively. The maximum change of Rx5day in the future is increased (up to 120 mm), with a positive trend of up to 1.6 mm/year for Epoch 3 under scenario SSP585.

**Figure S15**: **(a)** Change of the Rx1day (in mm) during three different future Epochs as compared to the reference period, derived from the MME of the 14 GCM models for four SSP scenarios (CMIP6). **(b)** Trend (in mm/year) of Rx1day during the reference period and three future Epochs. The green, purple, and white colors represent a statistically significant increasing, decreasing, and insignificant trend, respectively. The maximum change of Rx1day in the future is increased (up to 42mm), with a positive trend of up to 0.6 mm/year for Epoch 3 under scenario SSP585.

**Figure S16:** **(a)** Change of the R95P (in %) during three different future Epochs as compared to the reference period, derived from the MME of the 14 GCM models for four SSP scenarios (CMIP6). **(b)** Trend (in %/year) of R95P during the reference period and three future Epochs. The green, purple, and white color represent a statistically significant increasing, decreasing trend, and insignificant trend, respectively. The maximum change of R95p in the future is increased (up to 8%), with a positive trend of up to 0.085%/year for Epoch 3 under scenario SSP585.

**Figure S17:** **(a)** Change of the R99P (in %) during three different future Epochs as compared to the reference period, derived from the MME of the 14 GCM models for four SSP scenarios (CMIP6). **(b)** Trend (in %/year) of R99P during the reference period and three future Epochs. The green, purple, and white color represent a statistically significant increasing, decreasing trend, and insignificant trend, respectively. The maximum change of R99p in the future is increased (up to 5%), with a positive trend of up to 0.065%/year for Epoch 3 under scenario SSP585.

**Figure S18: (a)** Change of the R20mm (in Days) during three different future Epochs as compared to the reference period, derived from the MME of the 14 GCM models for four SSP scenarios (CMIP6). **(b)** Trend (in Days/year) of R20mm during the reference period and three future Epochs. The green, purple, and white color represent a statistically significant increasing, decreasing trend, and insignificant trend, respectively. The maximum change of R20mm in the future is increased (up to 14 days) with a positive trend of up to 0.3 days/year for Epoch 3 under scenario SSP585.

**Table T1:** Matrix of Pearson's correlation coefficients between the extreme climate indices for the reference and future period under four different scenarios with a 5% significance level. (The bold number indicates the significance at a 5% significance level)

| **Historical Period** | | | | | | | | | | | | |
| --- | --- | --- | --- | --- | --- | --- | --- | --- | --- | --- | --- | --- |
|  | **TXx** | **TXn** | **TNx** | **TNn** | **TX90p** | **TN90p** | **WSDI** | **SU** | **CSDI** | **TX10p** | **TN10p** | **DTR** |
| **CDD** | 0.13 | ***0.57*** | 0.06 | ***0.4*** | 0.23 | 0.14 | -0.25 | ***0.62*** | 0.15 | ***-0.61*** | ***-0.38*** | **0.33** |
| **CWD** | 0 | 0.16 | 0.22 | 0.17 | 0.1 | 0.2 | -0.16 | 0.04 | 0.08 | 0.05 | -0.22 | -0.23 |
| **R20** | -0.29 | -0.05 | ***-0.34*** | 0.03 | ***-0.43*** | -0.21 | 0.22 | -0.29 | ***-0.44*** | 0.22 | 0.17 | ***-0.34*** |
| **SDII** | -0.28 | 0.04 | ***-0.41*** | 0.04 | -0.26 | -0.29 | 0.16 | -0.03 | -0.26 | -0.02 | 0.05 | -0.05 |
| **Rx1** | 0.15 | 0.13 | -0.06 | 0.07 | -0.17 | 0.08 | ***0.34*** | -0.31 | -0.21 | 0.17 | 0.13 | -0.32 |
| **Rx5** | 0.21 | 0.06 | -0.15 | -0.03 | -0.15 | -0.02 | 0.32 | -0.25 | -0.17 | 0.15 | 0.17 | -0.18 |
| **R95p** | -0.01 | 0.04 | -0.02 | 0.02 | -0.04 | 0.09 | 0.17 | -0.12 | -0.04 | 0.04 | 0.01 | -0.16 |
| **R99P** | 0.06 | -0.28 | 0.13 | -0.16 | 0.08 | 0.25 | 0.28 | -0.17 | -0.11 | 0.08 | 0.1 | -0.07 |
| **Future Period (SSP126)** | | | | | | | | | | | | |
|  | **TXx** | **TXn** | **TNx** | **TNn** | **TX90p** | **TN90p** | **WSDI** | **SU** | **CSDI** | **TX10p** | **TN10p** | **DTR** |
| **CDD** | 0.02 | ***0.21*** | -0.02 | 0.1 | 0.1 | ***-0.22*** | -0.14 | -0.13 | 0.04 | 0.06 | -0.15 | ***0.27*** |
| **CWD** | -0.2 | 0.04 | ***-0.24*** | -0.18 | ***-0.3*** | ***-0.38*** | -0.17 | -0.13 | 0.18 | 0.07 | ***-0.16*** | 0.1 |
| **R20** | -0.3 | -0.03 | 0.16 | 0.12 | ***-0.38*** | -0.35 | ***0.37*** | -0.24 | -0.33 | 0.27 | 0.16 | ***-0.54*** |
| **SDII** | -0.1 | 0.17 | -0.21 | 0.1 | -0.2 | 0.21 | 0.18 | 0.02 | -0.14 | -0.15 | -0.18 | -0.19 |
| **Rx1** | -0.07 | 0.14 | 0.12 | -0.01 | -0.09 | 0.09 | -0.17 | 0.07 | -0.06 | -0.12 | -0.09 | -0.12 |
| **Rx5** | 0.03 | 0.16 | 0.15 | 0.08 | -0.08 | 0.09 | -0.13 | 0.06 | -0.14 | -0.14 | -0.16 | -0.18 |
| **R95p** | -0.04 | 0.18 | 0.08 | 0.14 | -0.08 | 0.11 | -0.05 | 0.1 | -0.19 | -0.17 | -0.19 | -0.11 |
| **R99P** | 0.01 | ***0.23*** | 0.17 | 0.09 | 0.03 | 0.15 | 0.15 | 0.12 | -0.16 | -0.19 | -0.15 | -0.01 |
| **Future Period (SSP245)** | | | | | | | | | | | | |
|  | **TXx** | **TXn** | **TNx** | **TNn** | **TX90p** | **TN90p** | **WSDI** | **SU** | **CSDI** | **TX10p** | **TN10p** | **DTR** |
| **CDD** | -0.03 | -0.01 | -0.14 | -0.08 | -0.09 | -0.17 | -0.07 | -0.01 | 0.19 | -0.03 | 0.13 | ***0.48*** |
| **CWD** | ***-0.24*** | ***-0.29*** | ***-0.23*** | ***-0.25*** | ***-0.23*** | ***-0.27*** | ***-0.12*** | ***-0.22*** | ***0.24*** | ***0.23*** | 0.23 | -0.16 |
| **R20** | ***-0.29*** | 0.18 | -0.11 | 0.18 | -0.18 | 0.09 | ***-0.23*** | 0.07 | -0.14 | -0.15 | -0.15 | ***-0.46*** |
| **SDII** | -0.1 | ***0.26*** | 0.09 | ***0.34*** | 0.05 | ***0.24*** | 0.03 | **0.25** | ***-0.28*** | ***-0.29*** | ***-0.3*** | ***-0.22*** |
| **Rx1** | 0.01 | 0.13 | ***0.32*** | ***0.27*** | 0.18 | ***0.33*** | 0.14 | 0.13 | ***-0.33*** | -0.21 | ***-0.31*** | ***-0.42*** |
| **Rx5** | -0.11 | 0.17 | ***0.16*** | ***0.23*** | 0.02 | ***0.23*** | 0 | 0.1 | ***-0.26*** | -0.16 | ***-0.26*** | ***-0.41*** |
| **R95p** | 0.06 | 0.21 | ***0.21*** | ***0.29*** | 0.09 | ***0.28*** | 0.09 | 0.17 | ***-0.26*** | **-0.23** | ***-0.28*** | ***-0.24*** |
| **R99P** | 0.1 | 0.19 | ***0.34*** | ***0.31*** | 0.2 | ***0.34*** | 0.17 | 0.18 | ***-0.36*** | -0.2 | ***-0.35*** | ***-0.32*** |

| **Future Period (SSP370)** | | | | | | | | | | | | |
| --- | --- | --- | --- | --- | --- | --- | --- | --- | --- | --- | --- | --- |
|  | **TXx** | **TXn** | **TNx** | **TNn** | **TX90p** | **TN90p** | **WSDI** | **SU** | **CSDI** | **TX10p** | **TN10p** | **DTR** |
| **CDD** | -0.02 | ***0.25*** | -0.06 | 0.04 | -0.07 | -0.04 | 0.11 | 0.12 | -0.01 | -0.2 | 0.01 | ***0.38*** |
| **CWD** | 0.07 | -0.03 | 0 | -0.03 | 0.02 | -0.15 | ***0.23*** | -0.08 | 0.04 | 0.12 | 0.2 | 0.11 |
| **R20** | -0.02 | ***0.28*** | ***0.34*** | ***0.46*** | ***0.36*** | ***0.44*** | ***0.31*** | ***0.35*** | ***-0.41*** | ***-0.28*** | ***-0.41*** | ***-0.57*** |
| **SDII** | ***0.46*** | ***0.46*** | ***0.58*** | ***0.59*** | ***0.61*** | ***0.63*** | ***0.59*** | ***0.56*** | ***-0.53*** | ***-0.46*** | ***-0.52*** | ***-0.41*** |
| **Rx1** | ***0.34*** | ***0.37*** | ***0.5*** | ***0.52*** | ***0.51*** | ***0.53*** | ***0.51*** | ***0.44*** | ***-0.45*** | ***-0.34*** | ***-0.44*** | ***-0.47*** |
| **Rx5** | ***0.34*** | ***0.43*** | ***0.51*** | ***0.53*** | ***0.54*** | ***0.55*** | ***0.51*** | ***0.48*** | ***-0.47*** | ***-0.37*** | ***-0.44*** | ***-0.4*** |
| **R95p** | ***0.54*** | ***0.48*** | ***0.68*** | ***0.61*** | ***0.69*** | ***0.71*** | ***0.63*** | ***0.61*** | ***-0.58*** | ***-0.49*** | ***-0.57*** | ***-0.44*** |
| **R99P** | ***0.57*** | ***0.53*** | ***0.74*** | ***0.63*** | ***0.74*** | ***0.73*** | ***0.7*** | ***0.64*** | ***-0.64*** | ***-0.53*** | ***-0.59*** | ***-0.41*** |
| **Future Period (SSP585)** | | | | | | | | | | | | |
|  | **TXx** | **TXn** | **TNx** | **TNn** | **TX90p** | **TN90p** | **WSDI** | **SU** | **CSDI** | **TX10p** | **TN10p** | **DTR** |
| **CDD** | 0.36 | 0.72 | ***-0.23*** | ***-0.25*** | ***-0.25*** | 0.07 | 0.15 | 0.17 | ***0.28*** | 0.97 | 0.12 | ***0.41*** |
| **CWD** | 0.94 | 0.95 | 0.7 | 0.77 | 0.86 | 0.1 | ***0.26*** | 0.11 | ***0.23*** | 0.27 | 0.27 | 0.26 |
| **R20** | ***0.39*** | ***0.41*** | ***0.55*** | ***0.55*** | ***0.53*** | ***0.55*** | ***0.44*** | ***0.49*** | ***-0.49*** | ***-0.37*** | ***-0.44*** | ***-0.58*** |
| **SDII** | ***0.55*** | ***0.52*** | ***0.66*** | ***0.62*** | ***0.65*** | ***0.63*** | ***0.61*** | ***0.57*** | ***-0.55*** | ***-0.45*** | ***-0.5*** | ***-0.49*** |
| **Rx1** | ***0.48*** | ***0.52*** | ***0.62*** | ***0.63*** | ***0.61*** | ***0.61*** | ***0.55*** | ***0.52*** | ***-0.55*** | ***-0.43*** | ***-0.48*** | ***-0.53*** |
| **Rx5** | ***0.44*** | ***0.45*** | ***0.59*** | ***0.59*** | ***0.58*** | ***0.55*** | ***0.54*** | ***0.45*** | ***-0.49*** | ***-0.34*** | ***-0.44*** | ***-0.55*** |
| **R95p** | ***0.61*** | ***0.52*** | ***0.69*** | ***0.63*** | ***0.67*** | ***0.67*** | ***0.64*** | ***0.58*** | ***-0.58*** | ***-0.47*** | ***-0.55*** | ***-0.5*** |
| **R99P** | ***0.64*** | ***0.56*** | ***0.74*** | ***0.67*** | ***0.72*** | ***0.68*** | ***0.73*** | ***0.56*** | ***-0.57*** | ***-0.44*** | ***-0.52*** | ***-0.5*** |

|  |  | ***-6*** |  |  | ***0*** |  |  | ***6*** | ***8*** | ***10*** |
| --- | --- | --- | --- | --- | --- | --- | --- | --- | --- | --- |
| **-1** | **-0.8** | **-0.6** | **-0.4** | **-0.2** | **0** | **0.2** | **0.4** | **0.6** | **0.8** | **1** |

| **a)** | Reference Period  (1981-2014) | Scenarios | Future Period | | |
| --- | --- | --- | --- | --- | --- |
|  |  |  | Epoch 1 | Epoch 2 | Epoch 3 |
| **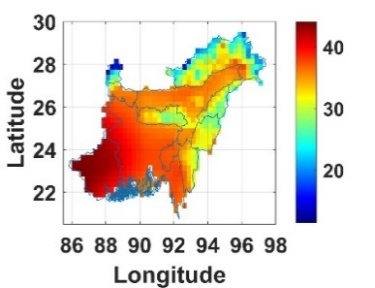** | | SSP126 | 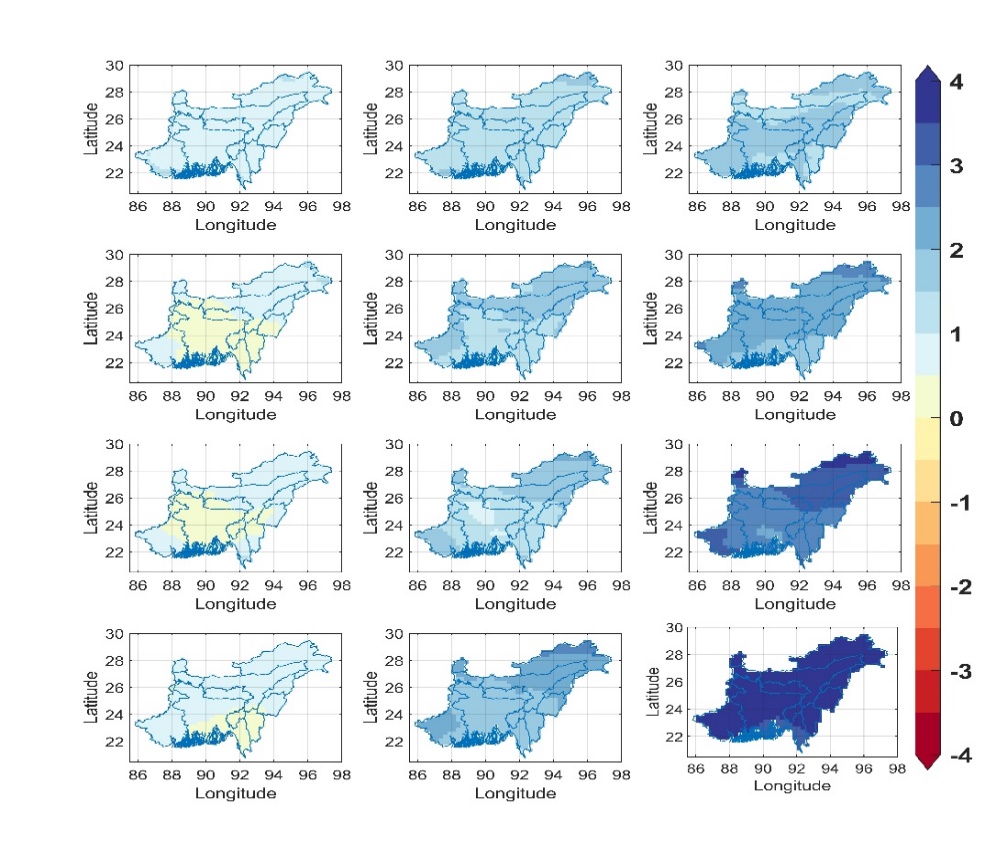 | | |
|  |  | SSP245 |  |  |  |
|  |  | SSP370 |  |  |  |
|  |  | SSP585 |  |  |  |
| **b)** | Reference Period |  | Epoch 1 | Epoch 2 | Epoch 3 |
| **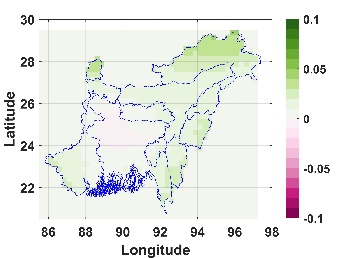** | | SSP126 | 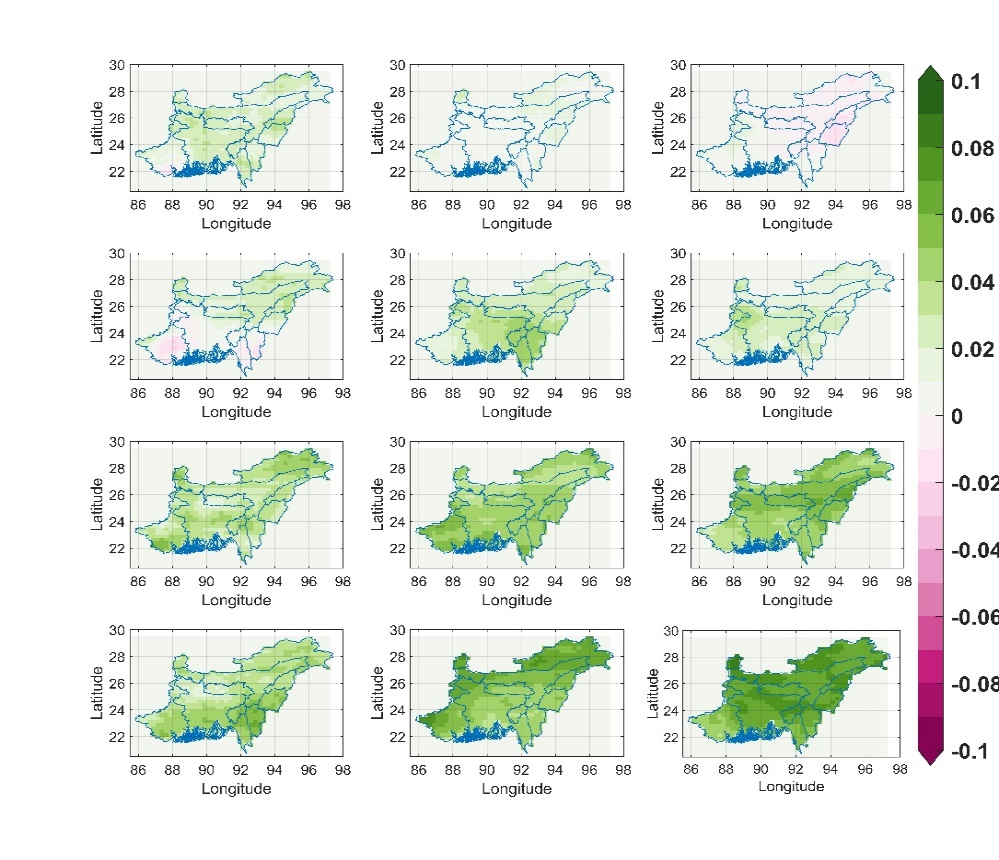 | | |
|  |  | SSP245 |  |  |  |
|  |  | SSP370 |  |  |  |
|  |  | SSP585 |  |  |  |

**Figure S1**: **(a)** Change of the TXx (in °C) during three different future Epochs as compared to the reference period, derived from the MME of the 14 GCM models for four SSP scenarios (CMIP6). **(b)** Trend (in °C/year) of TXx, during the reference period and three different future Epochs. The green, purple, and white color represent a statistically significant increasing, decreasing trend, and insignificant trend, respectively. The maximum change of TXx in the future is increased (up to 5°C), with a positive trend of up to 0.065°C/year for Epoch 3 under scenario SSP585.

| **a)** | Reference Period  (1981-2014) | Scenarios | Future Period | | |
| --- | --- | --- | --- | --- | --- |
|  |  |  | Epoch 1 | Epoch 2 | Epoch 3 |
| **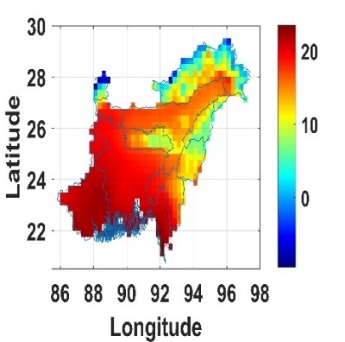** | | SSP126 | 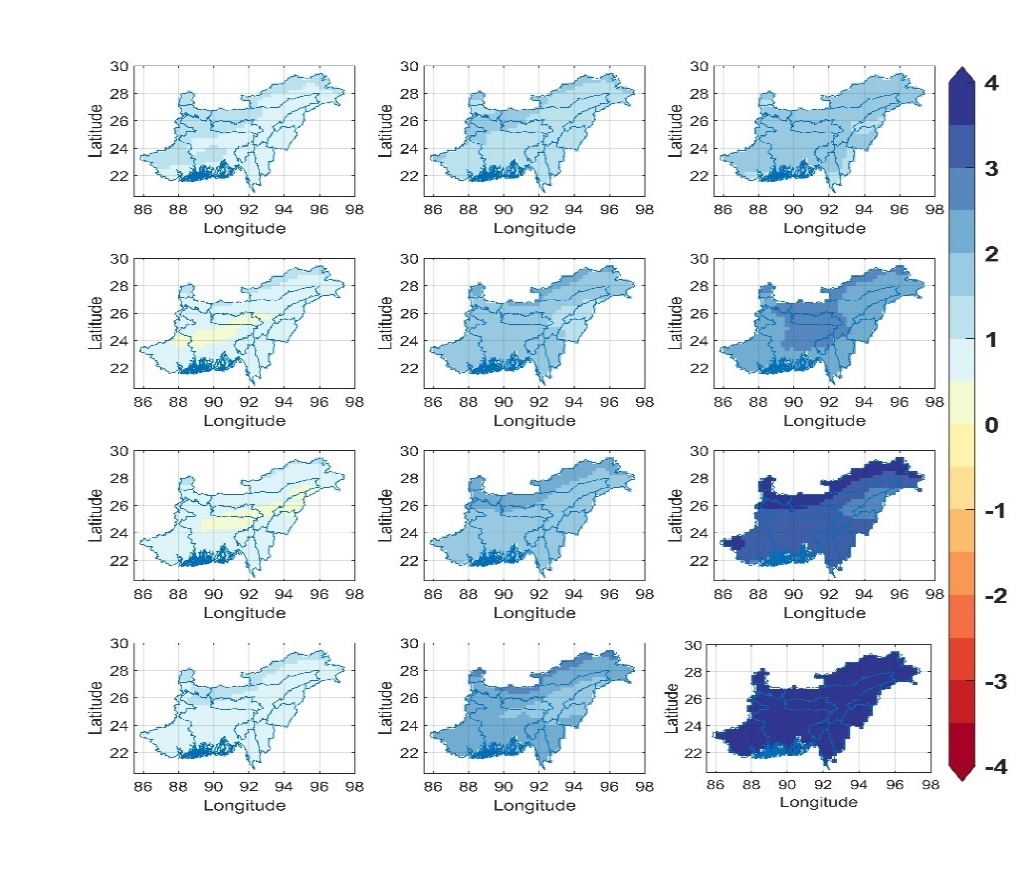 | | |
|  |  | SSP245 |  |  |  |
|  |  | SSP370 |  |  |  |
|  |  | SSP585 |  |  |  |
| **b)** | Reference Period |  | Epoch 1 | Epoch 2 | Epoch 3 |
| 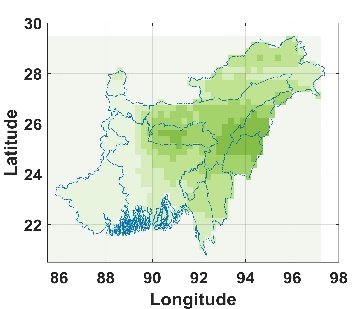 | | SSP126 | 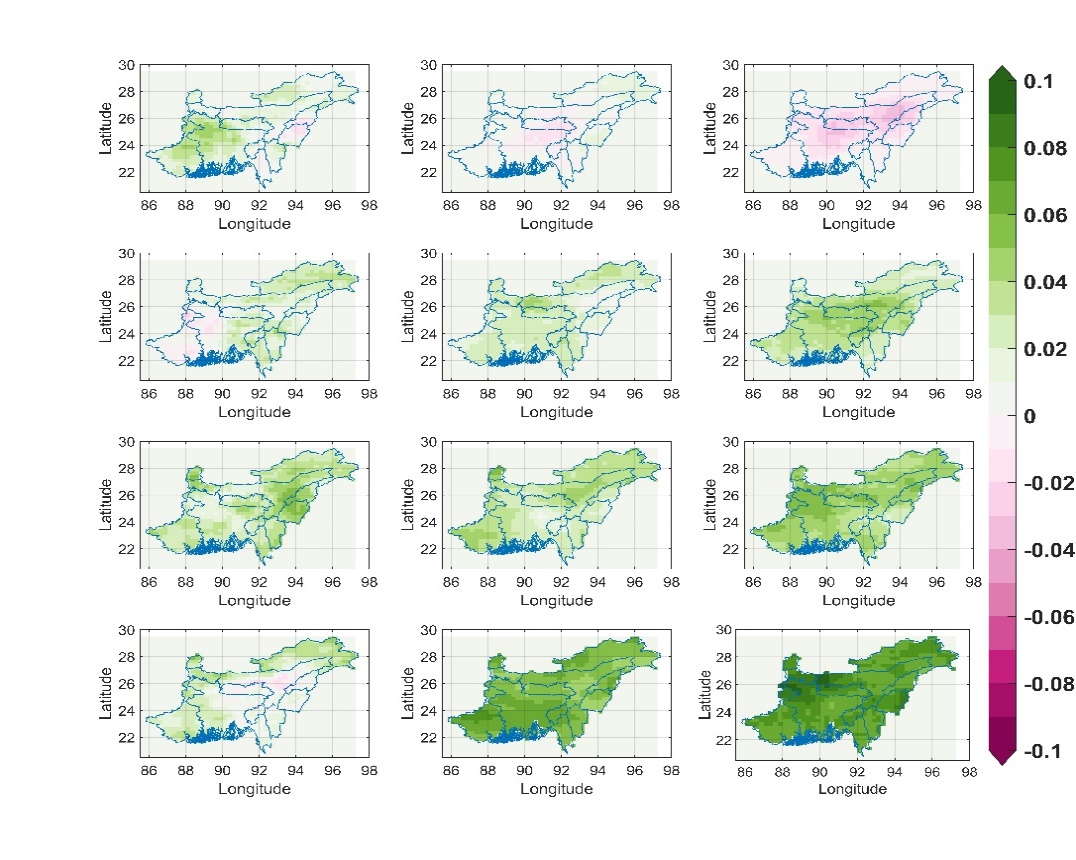 | | |
|  |  | SSP245 |  |  |  |
|  |  | SSP370 |  |  |  |
|  |  | SSP585 |  |  |  |

**Figure S2**: **(a)** Change of the TXn (in °C) during three different future Epochs as compared to the reference period, derived from the MME of the 14 GCM models for four SSP scenarios (CMIP6). **(b)** Trend (in °C/year) of TXn, during the reference period and three future Epochs. The green, purple, and white color represent a statistically significant increasing, decreasing trend, and insignificant trend, respectively. The maximum change of TXn in the future is increased (up to 5.5°C), with a positive trend of up to 0.06°C/year for Epoch 3 under scenario SSP585.

| **a)** | Reference Period  (1981-2014) | Scenarios | Future Period | | |
| --- | --- | --- | --- | --- | --- |
|  |  |  | Epoch 1 | Epoch 2 | Epoch 3 |
| **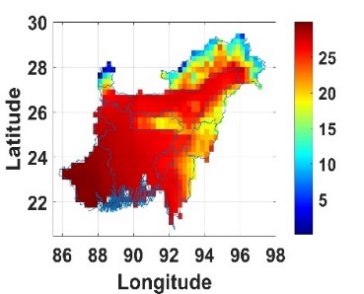** | | SSP126 | 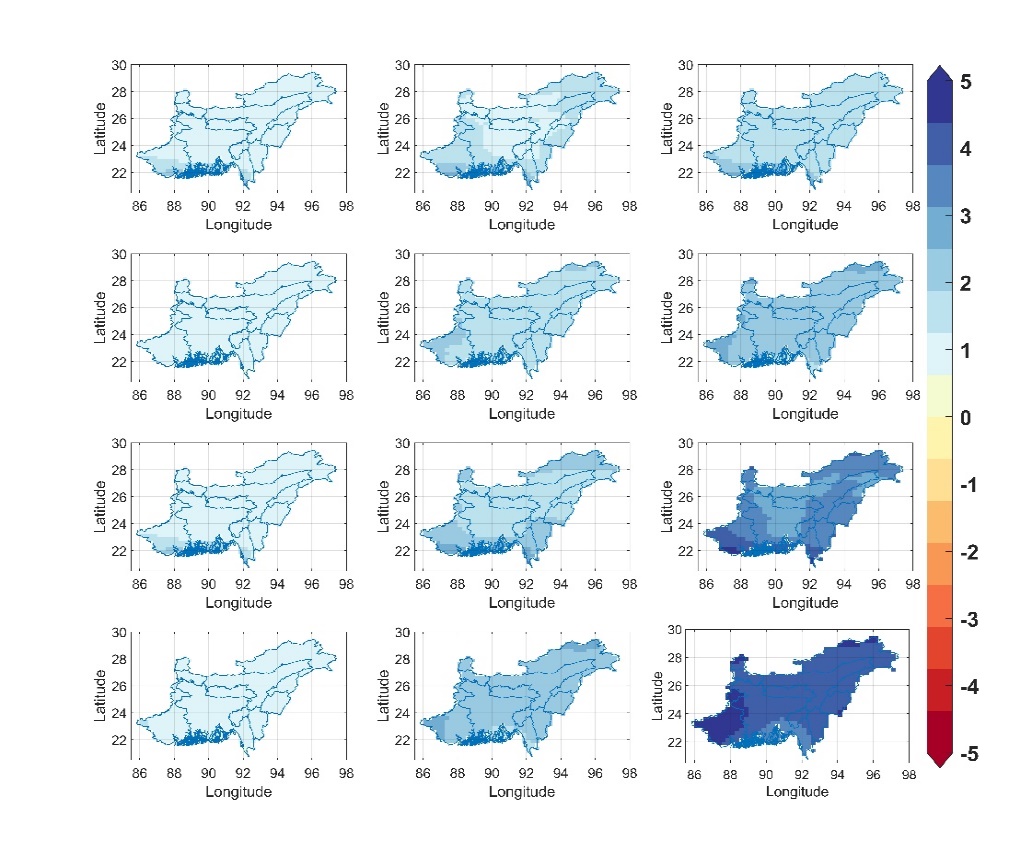 | | |
|  |  | SSP245 |  |  |  |
|  |  | SSP370 |  |  |  |
|  |  | SSP585 |  |  |  |
| **b)** | Reference Period |  | Epoch 1 | Epoch 2 | Epoch 3 |
| 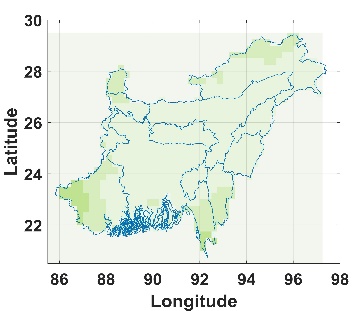 | | SSP126 | 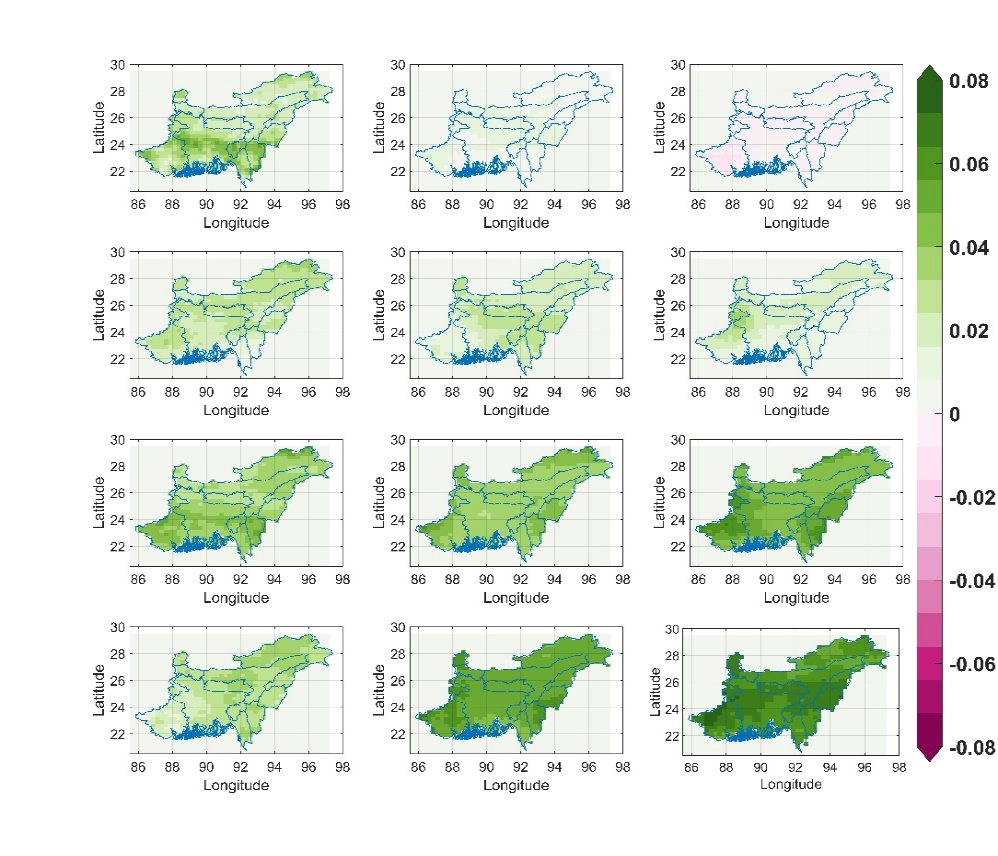 | | |
|  |  | SSP245 |  |  |  |
|  |  | SSP370 |  |  |  |
|  |  | SSP585 |  |  |  |

**Figure S3**: **(a)** Change of the TNx (in °C) during three different future Epochs as compared to the reference period, derived from the MME of the 14 GCM models for four SSP scenarios (CMIP6). **(b)** Trend (in °C/year) of TNx during the reference period and three different future Epochs. The green, purple, and white color represent a statistically significant increasing, decreasing trend, and insignificant trend, respectively. The maximum change of TNx in the future is increased (up to 5.2°C), with a positive trend of up to 0.05°C/year for Epoch 3 under scenario SSP585.

| **a)** | Reference Period  (1981-2014) | Scenarios | Future Period | | |
| --- | --- | --- | --- | --- | --- |
|  |  |  | Epoch 1 | Epoch 2 | Epoch 3 |
| **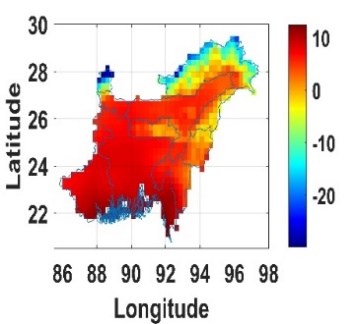** | | SSP126 | 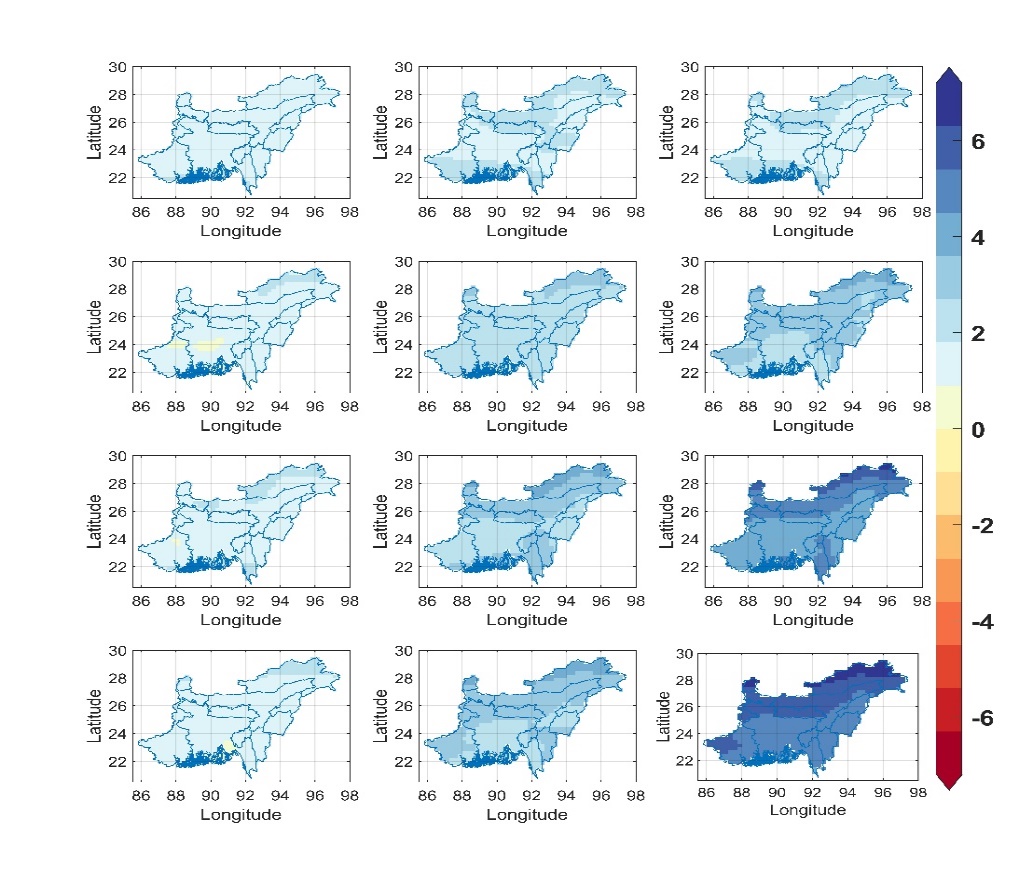 | | |
|  |  | SSP245 |  |  |  |
|  |  | SSP370 |  |  |  |
|  |  | SSP585 |  |  |  |
| **b)** | Reference Period |  | Epoch 1 | Epoch 2 | Epoch 3 |
| 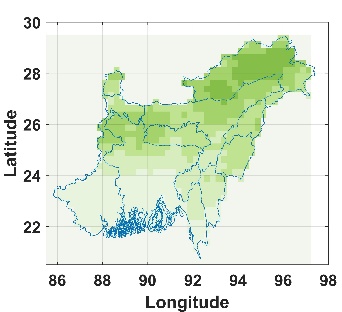 | | SSP126 | 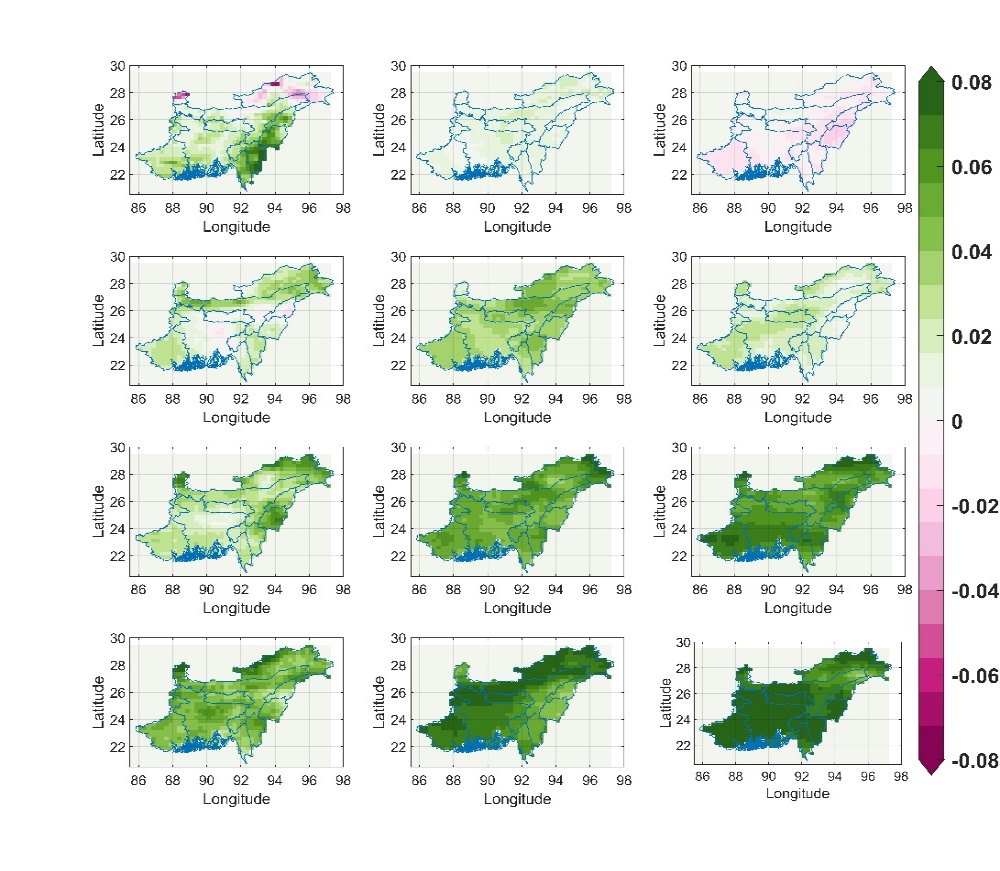 | | |
|  |  | SSP245 |  |  |  |
|  |  | SSP370 |  |  |  |
|  |  | SSP585 |  |  |  |

**Figure S4:** **(a)** Change of the TNn (in °C) during three different future Epochs as compared to the reference period, derived from the MME of the 14 GCM models for four SSP scenarios (CMIP6). **(b)** Trend (in °C/year) of TNn during the reference period and three different future Epochs. The green, purple, and white color represent a statistically significant increasing, decreasing trend, and insignificant trend, respectively. The maximum change of TNn in the future is increased (up to 7.2°C), with a positive trend of up to 0.08°C/year for Epoch 3 under scenario SSP585.

| **a)** | Reference Period  (1981-2014) | Scenarios | Future Period | | |
| --- | --- | --- | --- | --- | --- |
|  |  |  | Epoch 1 | Epoch 2 | Epoch 3 |
| **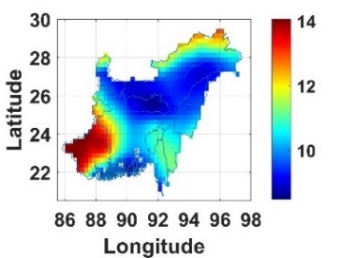** | | SSP126 | 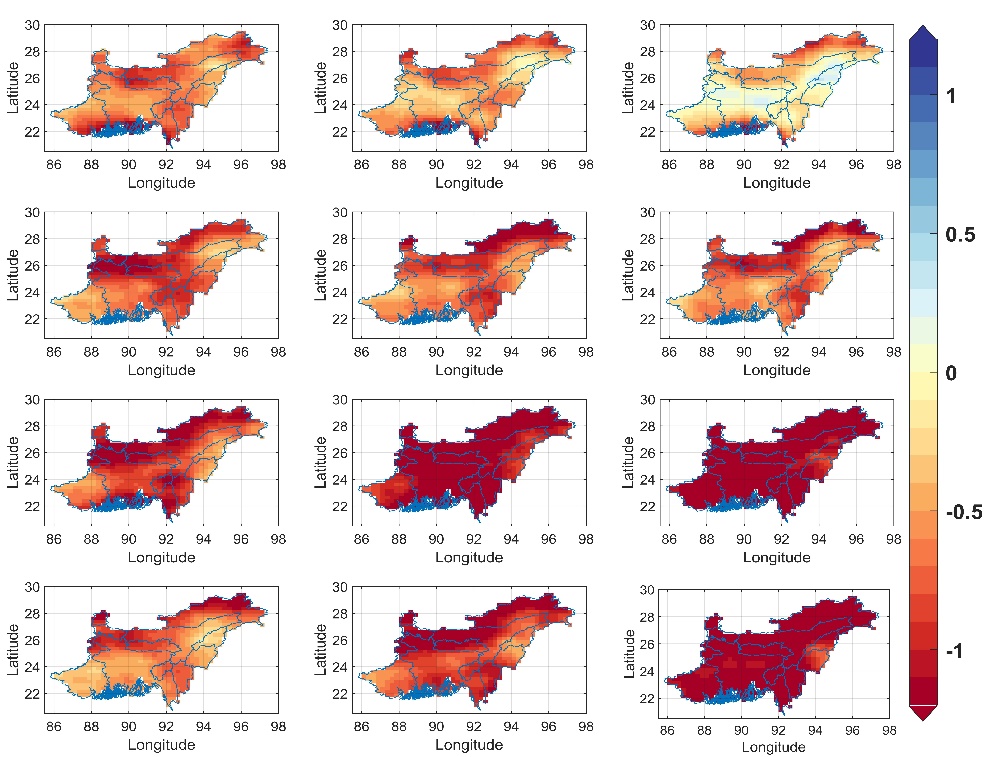 | | |
|  |  | SSP245 |  |  |  |
|  |  | SSP370 |  |  |  |
|  |  | SSP585 |  |  |  |
| **b)** | Reference Period |  | Epoch 1 | Epoch 2 | Epoch 3 |
| 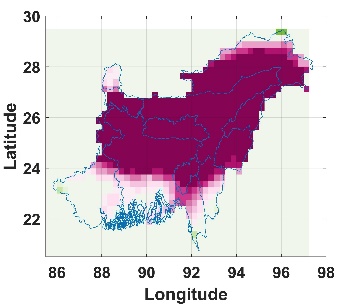 | | SSP126 | 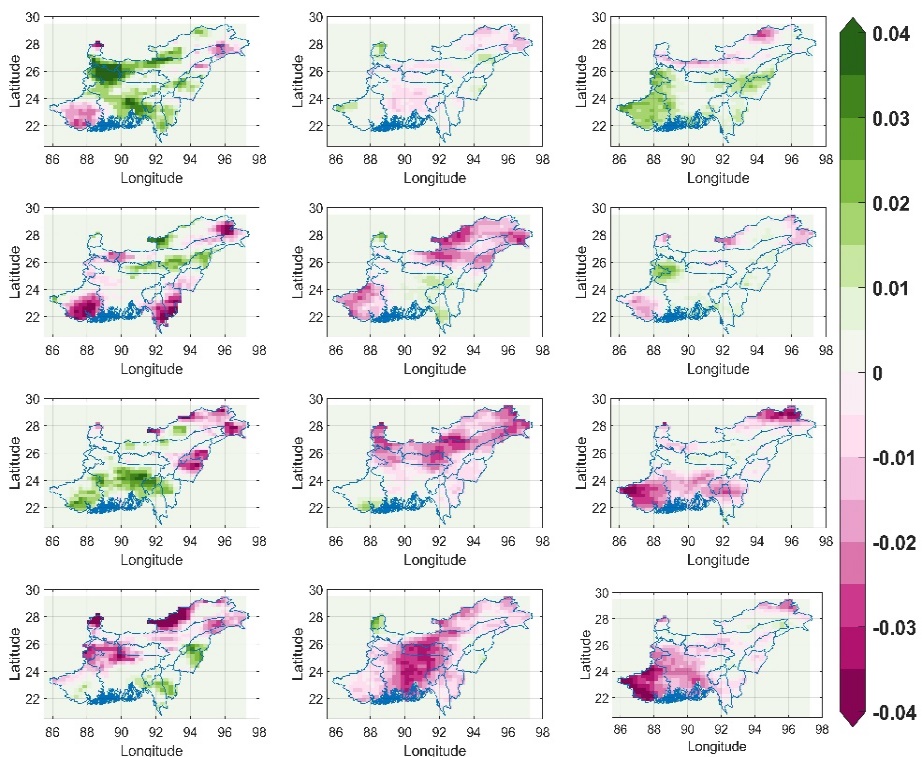 | | |
|  |  | SSP245 |  |  |  |
|  |  | SSP370 |  |  |  |
|  |  | SSP585 |  |  |  |

**Figure S5:** **(a)** Change of the DTR (in ˚C) during three different future Epochs as compared to the reference period, derived from the MME of the 14 GCM models for four SSP scenarios (CMIP6). **(b)** Trend (in ˚C/year) of DTR during the reference period and three different future Epochs. The green, purple, and white color represent a statistically significant increasing, decreasing trend, and insignificant trend, respectively. The maximum change of DTR in the future is decreased (up to -1.2 ˚C), mainly with a negative trend of up to -0.04 ˚C /year for Epoch 3 under scenario SSP585.

| **a)** | Reference Period  (1981-2014) | Scenarios | Future Period | | |
| --- | --- | --- | --- | --- | --- |
|  |  |  | Epoch 1 | Epoch 2 | Epoch 3 |
| **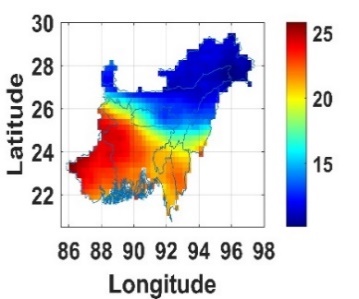** | | SSP126 | 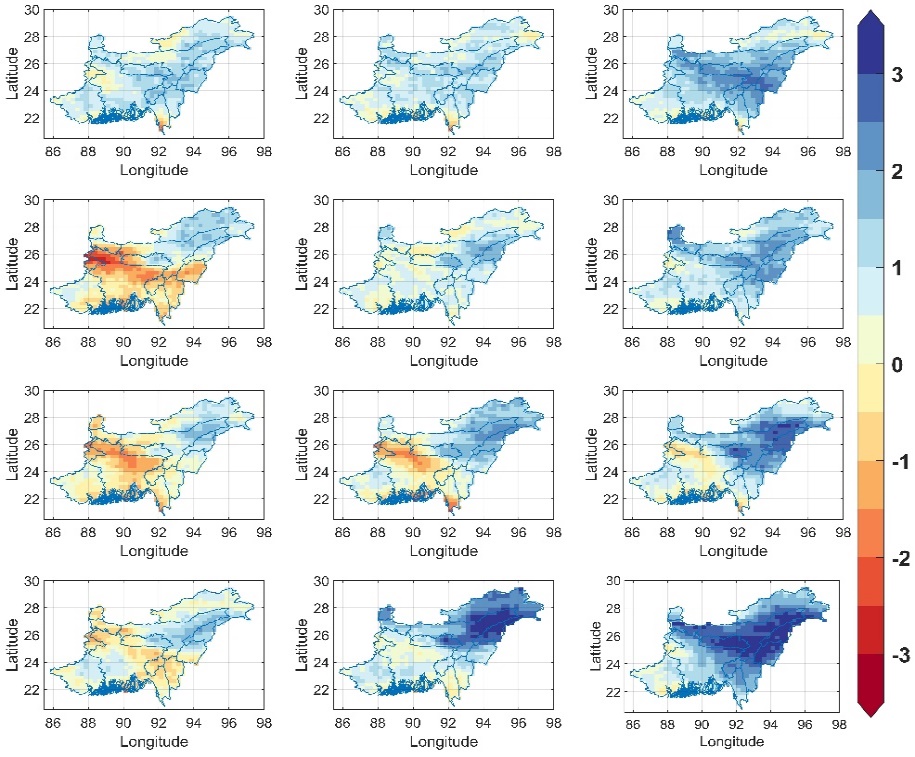 | | |
|  |  | SSP245 |  |  |  |
|  |  | SSP370 |  |  |  |
|  |  | SSP585 |  |  |  |
| **b)** | Reference Period |  | Epoch 1 | Epoch 2 | Epoch 3 |
| 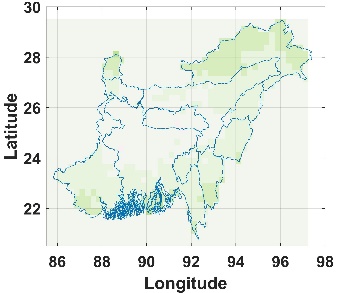 | | SSP126 | 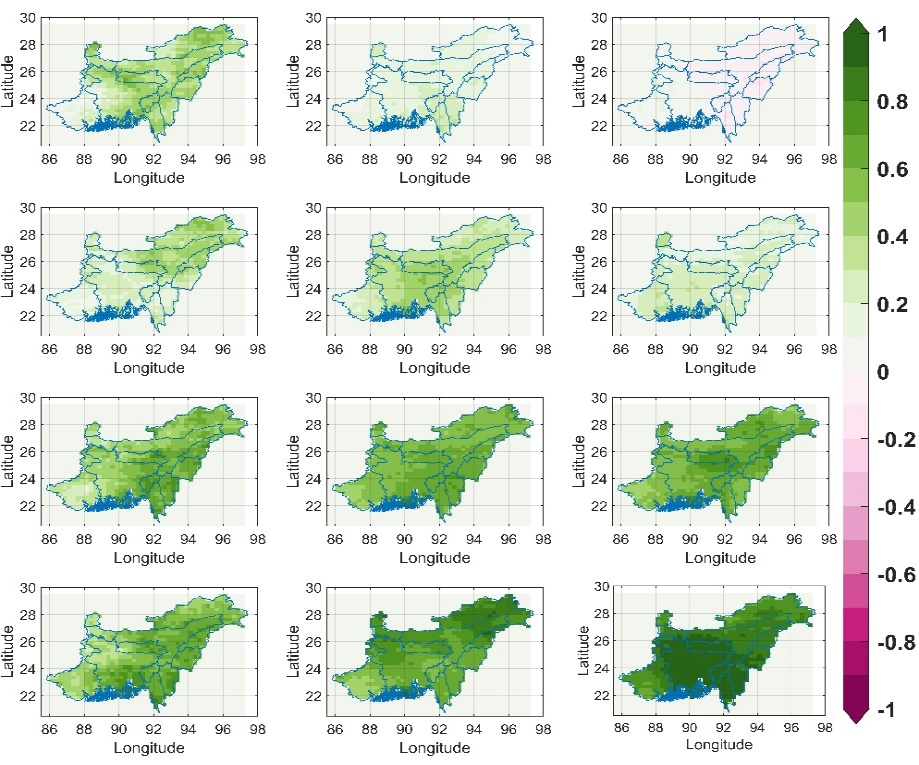 | | |
|  |  | SSP245 |  |  |  |
|  |  | SSP370 |  |  |  |
|  |  | SSP585 |  |  |  |

**Figure S6:** **(a)** Change of the WSDI (in Days) during three different future Epochs as compared to the reference period, derived from the MME of the 14 GCM models for four SSP scenarios (CMIP6). **(b)** Trend (in Days/year) of WSDI during the reference period and three future Epochs. The green, purple, and white color represent a statistically significant increasing, decreasing trend, and insignificant trend, respectively. The maximum change of WSDI in the future is increased (up to 3.5 Days), with a positive trend of up to 0.8 days/year for Epoch 3 under scenario SSP585.

| **a)** | Reference Period  (1981-2014) | Scenarios | Future Period | | |
| --- | --- | --- | --- | --- | --- |
|  |  |  | Epoch 1 | Epoch 2 | Epoch 3 |
| **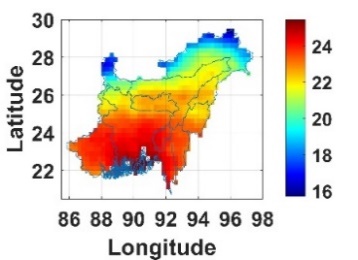** | | SSP126 | 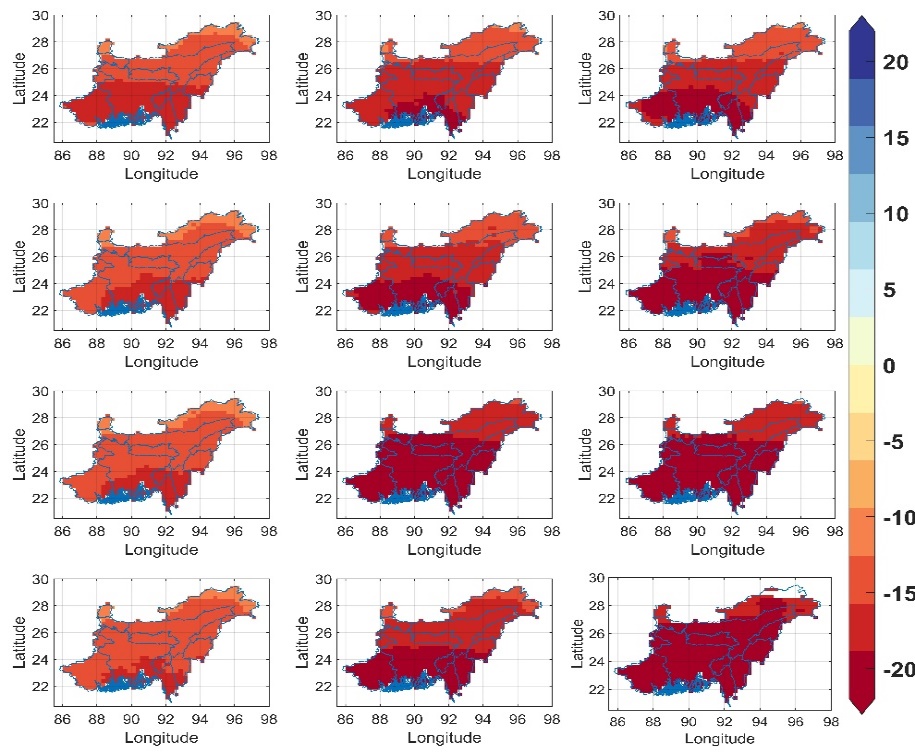 | | |
|  |  | SSP245 |  |  |  |
|  |  | SSP370 |  |  |  |
|  |  | SSP585 |  |  |  |
| **b)** | Reference Period |  | Epoch 1 | Epoch 2 | Epoch 3 |
| 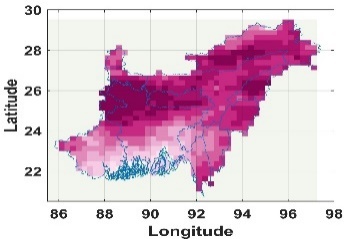 | | SSP126 | 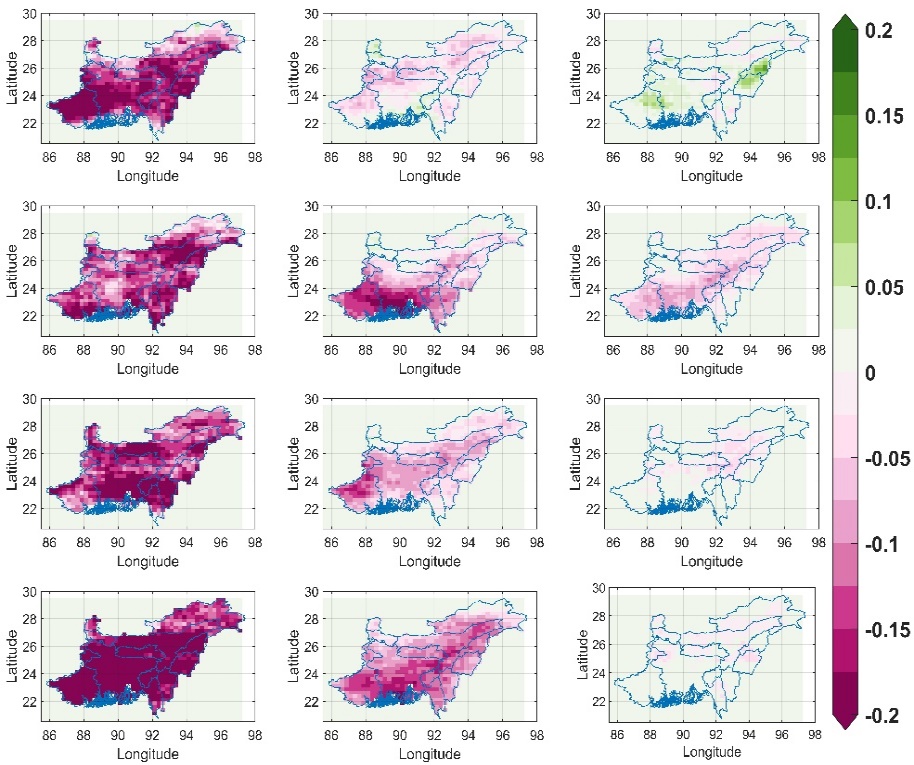 | | |
|  |  | SSP245 |  |  |  |
|  |  | SSP370 |  |  |  |
|  |  | SSP585 |  |  |  |

**Figure S7:** **(a)** Change of the CSDI (in Days) during three different future Epochs as compared to the reference period, derived from the MME of the 14 GCM models for four SSP scenarios (CMIP6). **(b)** Trend (in Days/year) of CSDI during the reference period and three future Epochs. The green, purple, and white colors represent a statistically significant increasing, decreasing, and insignificant trend, respectively. The maximum change of WSDI in the future is decreased (up to -22 Days), with a negative trend of up to -0.018 days/year for Epoch 3 under scenario SSP585.

| **a)** | Reference Period  (1981-2014) | | Scenarios | Future Period | | | | |
| --- | --- | --- | --- | --- | --- | --- | --- | --- |
|  |  |  |  | Epoch 1 | Epoch 2 | | | Epoch 3 |
| **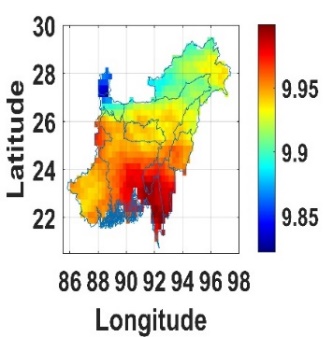** | | | SSP126 | 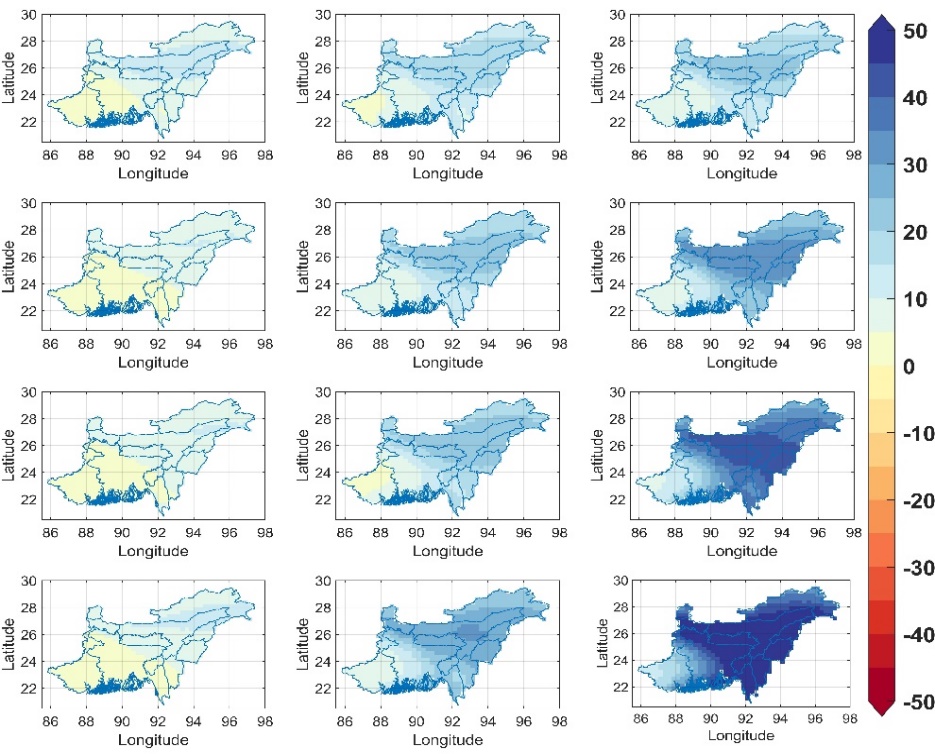 | | | | |
|  |  |  | SSP245 |  |  |  |  |  |
|  |  |  | SSP370 |  |  |  |  |  |
|  |  |  | SSP585 |  |  |  |  |  |
| **b)** | | Reference Period | | Epoch 1 | | Epoch 2 | Epoch 3 | |
| 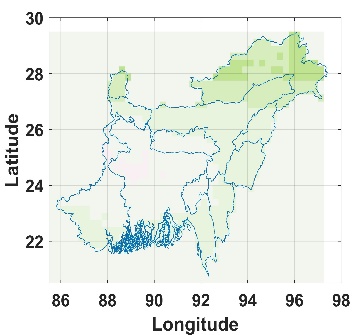 | | | SSP126 | 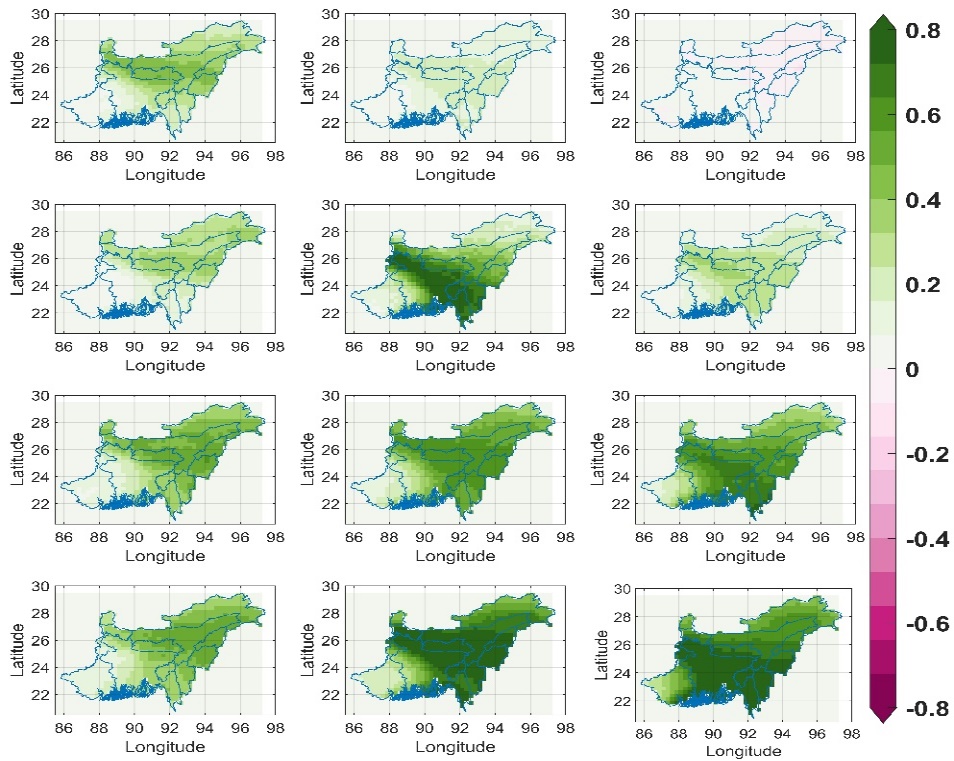 | | | | |
|  |  |  | SSP245 |  |  |  |  |  |
|  |  |  | SSP370 |  |  |  |  |  |
|  |  |  | SSP585 |  |  |  |  |  |

**Figure S8:** **(a)** Change of the TX90p (in %) during three different future Epochs as compared to the reference period, derived from the MME of the 14 GCM models for four SSP scenarios (CMIP6). **(b)** Trend (in %/year) of TX90p during the reference period and three future Epochs. The green, purple, and white color represent a statistically significant increasing, decreasing trend and insignificant. The maximum change of TX90p in the future is increased (up to 50 %), with a positive trend of up to 0.9 %/year for Epoch 3 under scenario SSP585.

| **a)** | Reference Period  (1981-2014) | Scenarios | Future Period | | |
| --- | --- | --- | --- | --- | --- |
|  |  |  | Epoch 1 | Epoch 2 | Epoch 3 |
| **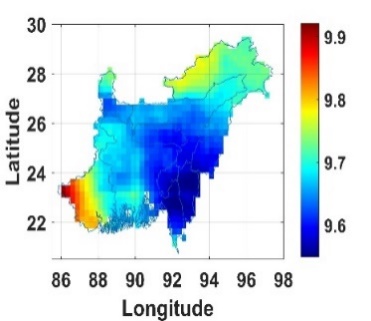** | | SSP126 | 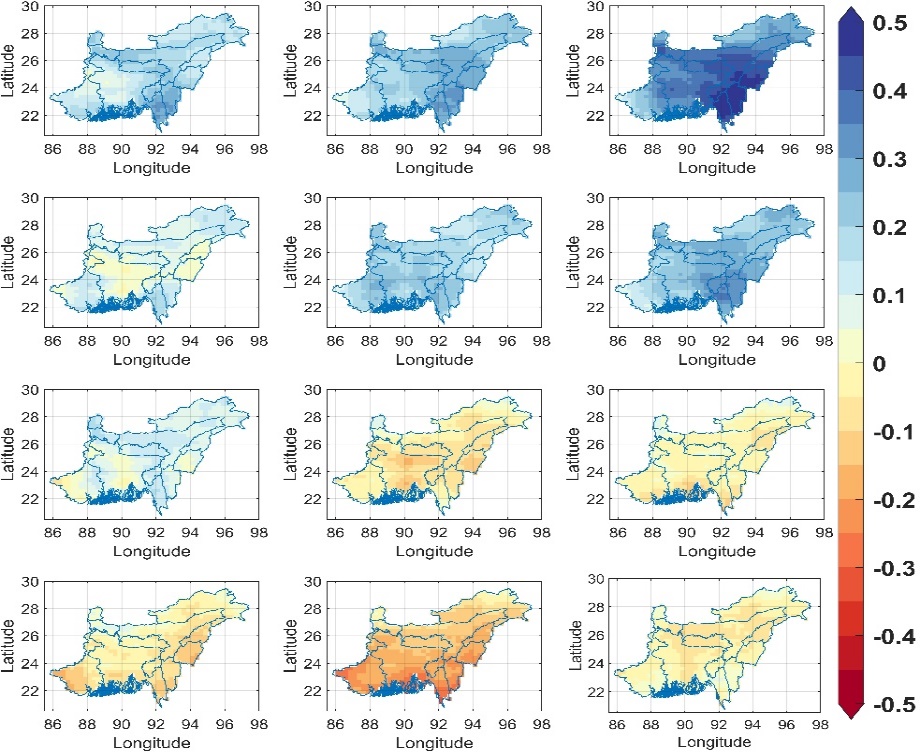 | | |
|  |  | SSP245 |  |  |  |
|  |  | SSP370 |  |  |  |
|  |  | SSP585 |  |  |  |
| **b)** | Reference Period |  | Epoch 1 | Epoch 2 | Epoch 3 |
| 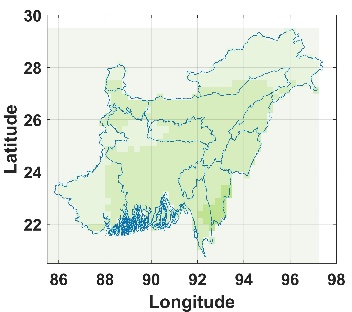 | | SSP126 | 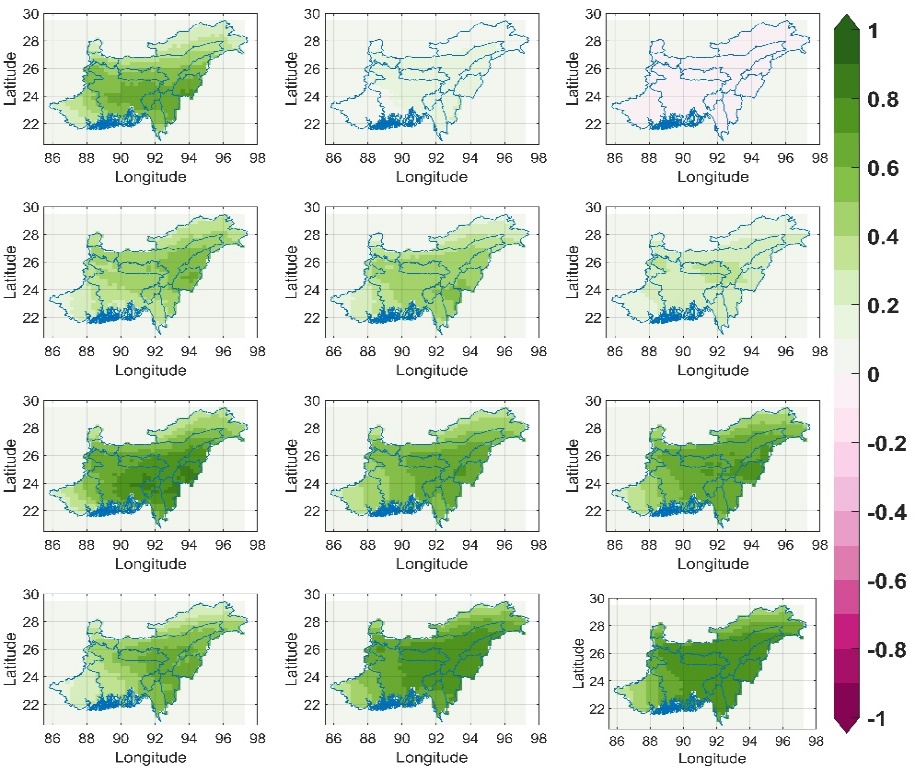 | | |
|  |  | SSP245 |  |  |  |
|  |  | SSP370 |  |  |  |
|  |  | SSP585 |  |  |  |

**Figure S9: (a)** Change of the TN90p (in %) during three different future Epochs as compared to the reference period, derived from the MME of the 14 GCM models for four SSP scenarios (CMIP6). **(b)** Trend (in %/year) of TN90p during the reference period and three future Epochs. The green, purple, and white color represent a statistically significant increasing, decreasing trend, and insignificant trend, respectively. The maximum change of TN90p in the future is decreased (up to -0.15 %), with a positive trend of up to 0.62 %/year for Epoch 3 under scenario SSP585.

| **a)** | Reference Period  (1981-2014) | | Scenarios | Future Period | | | | |
| --- | --- | --- | --- | --- | --- | --- | --- | --- |
|  |  |  |  | Epoch 1 | Epoch 2 | | | Epoch 3 |
| **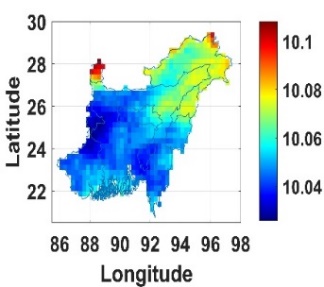** | | | SSP126 | 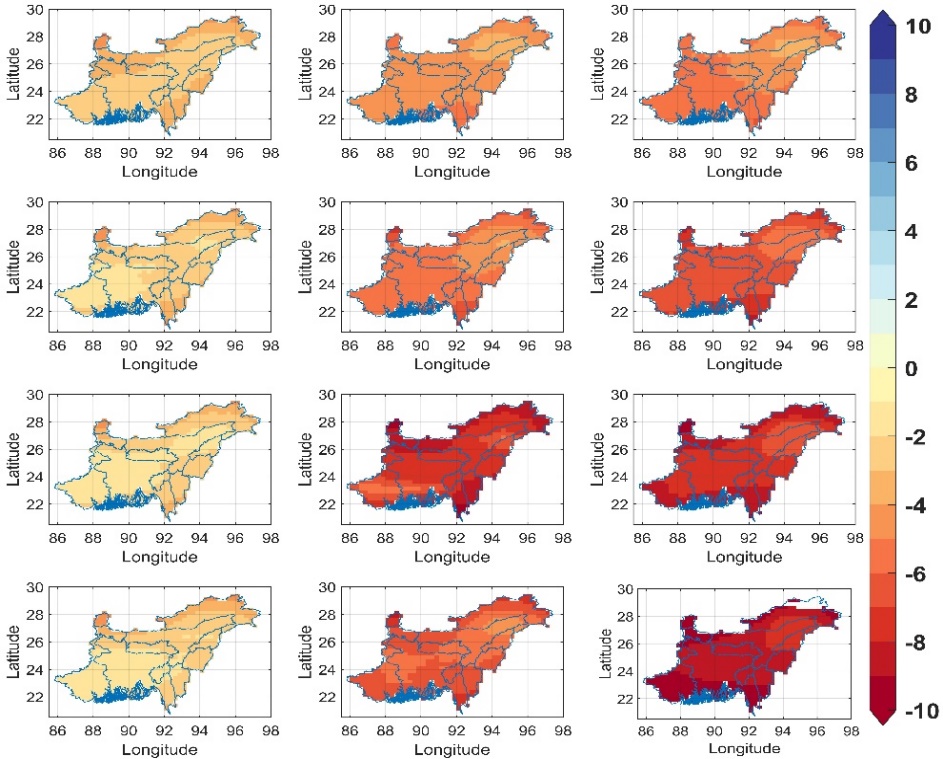 | | | | |
|  |  |  | SSP245 |  |  |  |  |  |
|  |  |  | SSP370 |  |  |  |  |  |
|  |  |  | SSP585 |  |  |  |  |  |
| **b)** | | Reference Period | | Epoch 1 | | Epoch 2 | Epoch 3 | |
| 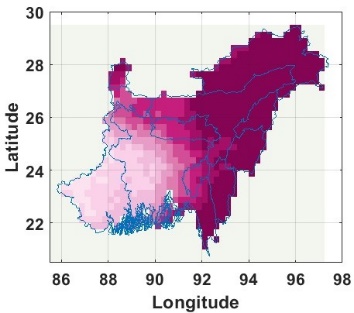 | | | SSP126 | 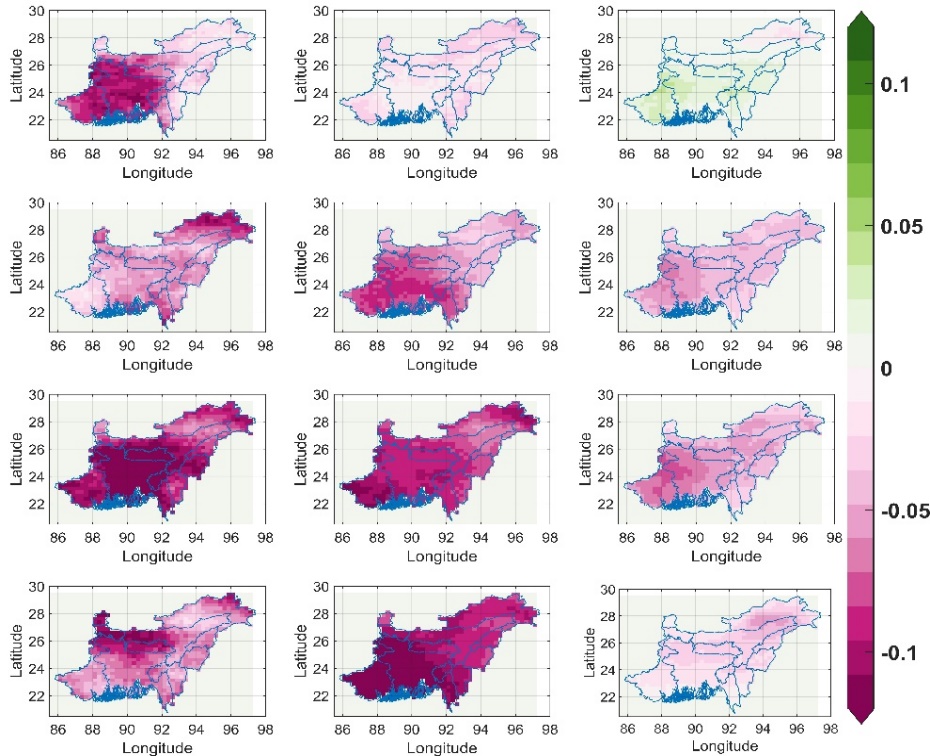 | | | | |
|  |  |  | SSP245 |  |  |  |  |  |
|  |  |  | SSP370 |  |  |  |  |  |
|  |  |  | SSP585 |  |  |  |  |  |

**Figure S10:** **(a)** Change of the TX10p (in %) during three different future Epochs as compared to the reference period, derived from the MME of the 14 GCM models for four SSP scenarios (CMIP6). **(b)** Trend (in %/year) of TX10p during the reference period and three future Epochs. The green, purple, and white color represent a statistically significant increasing, decreasing trend, and insignificant trend, respectively. The maximum change of TX10p in the future is decreased (up to -9.6 %), with a decreasing trend of up to -0.05 %/year for Epoch 3 under scenario SSP585.

| **a)** | Reference Period  (1981-2014) | Scenarios | Future Period | | |
| --- | --- | --- | --- | --- | --- |
|  |  |  | Epoch 1 | Epoch 2 | Epoch 3 |
| **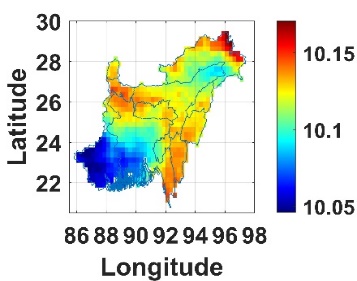** | | SSP126 | 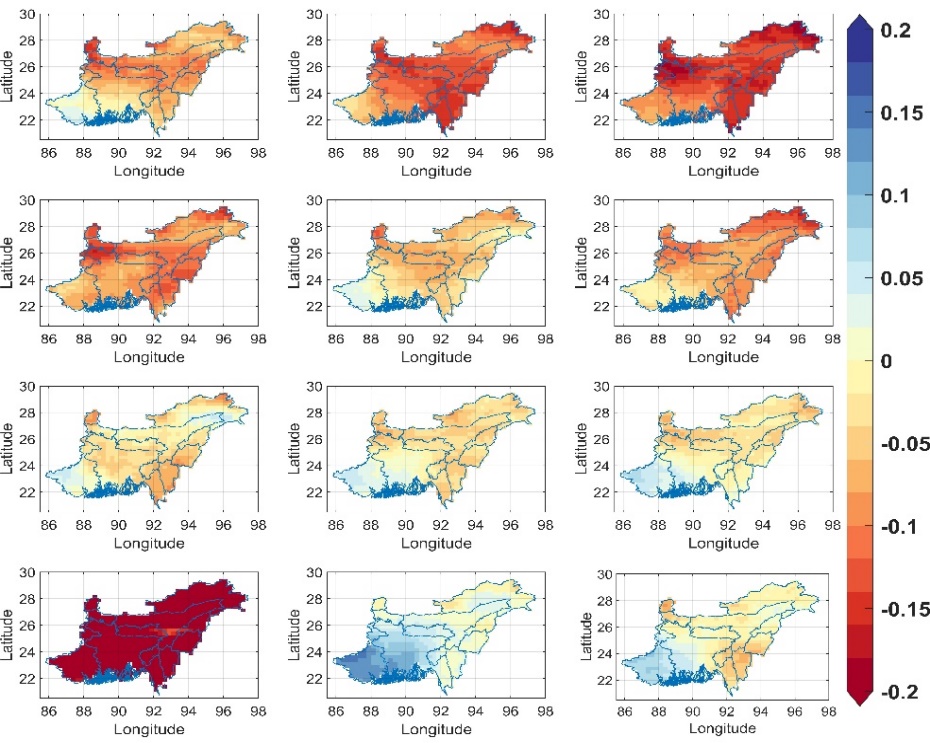 | | |
|  |  | SSP245 |  |  |  |
|  |  | SSP370 |  |  |  |
|  |  | SSP585 |  |  |  |
| **b)** | Reference Period |  | Epoch 1 | Epoch 2 | Epoch 3 |
| 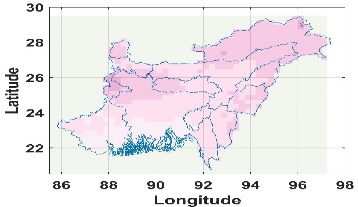 | | SSP126 | 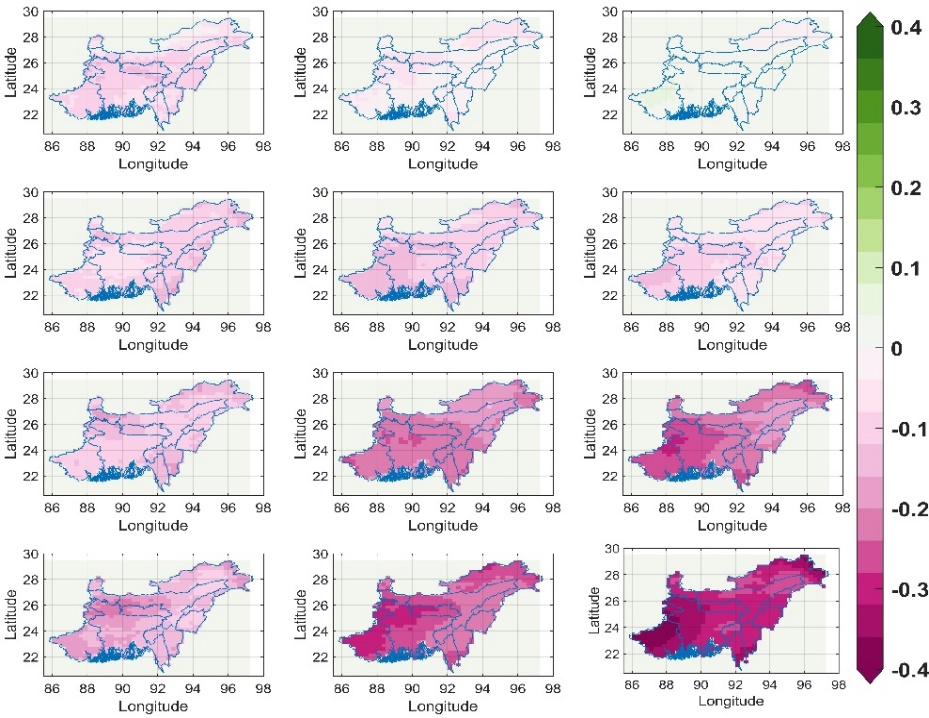 | | |
|  |  | SSP245 |  |  |  |
|  |  | SSP370 |  |  |  |
|  |  | SSP585 |  |  |  |

**Figure S11:** **(a)** Change of the TN10p (in %) during three different future Epochs as compared to the reference period, derived from the MME of the 14 GCM models for four SSP scenarios (CMIP6). **(b)** Trend (in %/year) of TN10p during the reference period and three future Epochs. The green, purple, and white color represent a statistically significant increasing, decreasing trend, and insignificant trend, respectively. The maximum change of TN10p in the future is both increasing (up to 0.065%) and decreasing (up to -0.07 %) in nature, with a negative trend of up to -0.35 %/year for Epoch 3 under scenario SSP585.

| **a)** | Reference Period  (1981-2014) | | Scenarios | Future Period | | | | |
| --- | --- | --- | --- | --- | --- | --- | --- | --- |
|  |  |  |  | Epoch 1 | Epoch 2 | | | Epoch 3 |
| **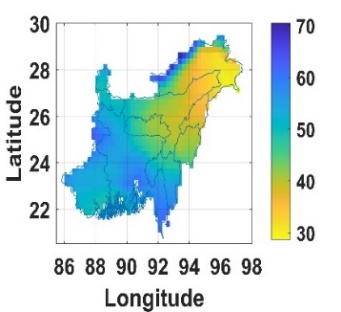** | | | SSP126 | 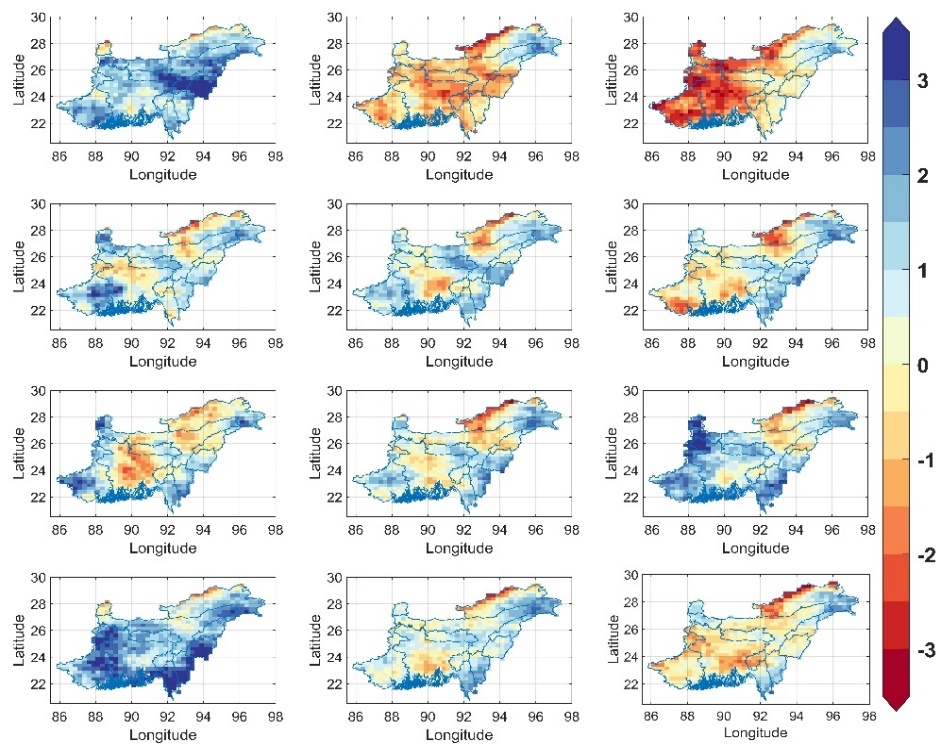 | | | | |
|  |  |  | SSP245 |  |  |  |  |  |
|  |  |  | SSP370 |  |  |  |  |  |
|  |  |  | SSP585 |  |  |  |  |  |
| **b)** | | Reference Period | | Epoch 1 | | Epoch 2 | Epoch 3 | |
| 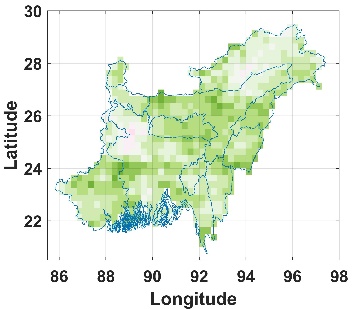 | | | SSP126 | 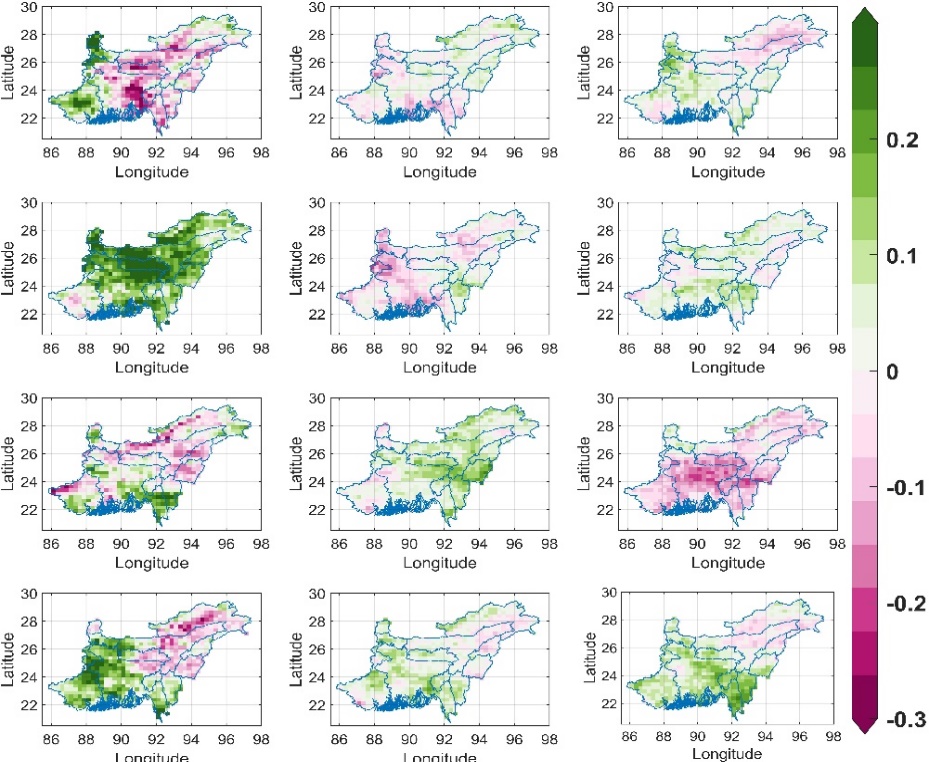 | | | | |
|  |  |  | SSP245 |  |  |  |  |  |
|  |  |  | SSP370 |  |  |  |  |  |
|  |  |  | SSP585 |  |  |  |  |  |

**Figure S12:** **(a)** Change of the CDD (in Days) during three different future Epochs as compared to the reference period, derived from the MME of the 14 GCM models for four SSP scenarios (CMIP6). **(b)** Trend (in Days/year) of CDD during the reference period and three future Epochs. The green, purple, and white color represent a statistically significant increasing, decreasing trend, and insignificant trend, respectively. The maximum change of CDD in the future is increased (up to 2 days) and decreasing (up to -3.5 days) in nature, with a mixed trend for Epoch3 under scenario SSP585.

| **a)** | Reference Period  (1981-2014) | | Scenarios | Future Period | | | | |
| --- | --- | --- | --- | --- | --- | --- | --- | --- |
|  |  |  |  | Epoch 1 | Epoch 2 | | | Epoch 3 |
| **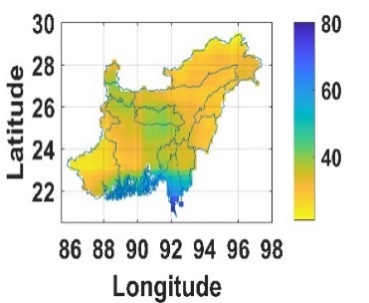** | | | SSP126 | 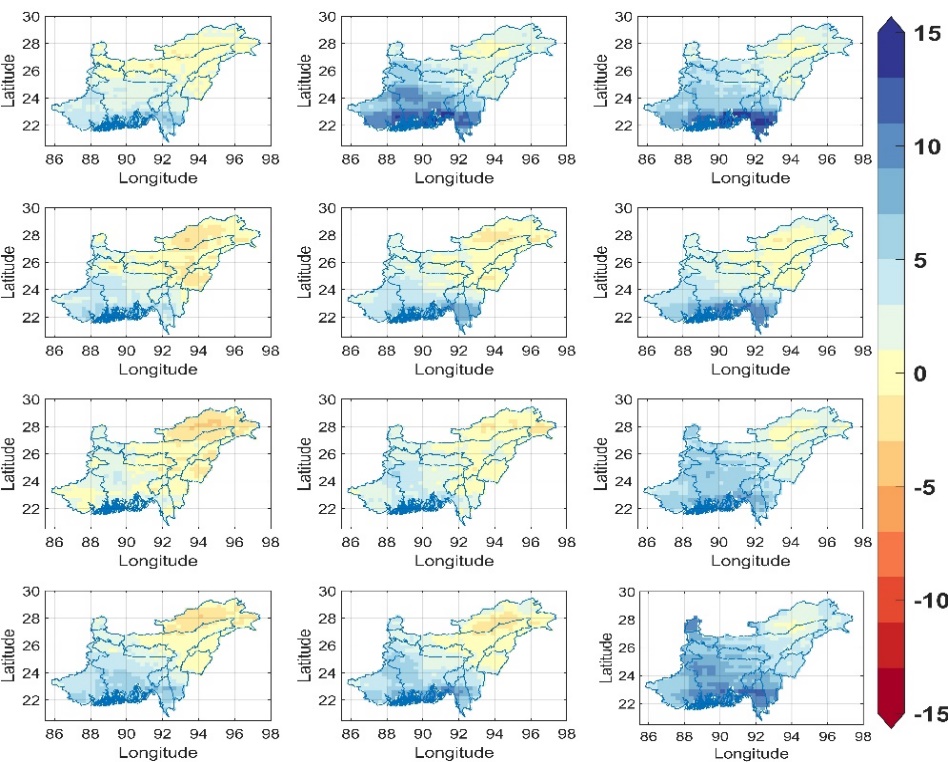 | | | | |
|  |  |  | SSP245 |  |  |  |  |  |
|  |  |  | SSP370 |  |  |  |  |  |
|  |  |  | SSP585 |  |  |  |  |  |
| **b)** | | Reference Period | | Epoch 1 | | Epoch 2 | Epoch 3 | |
| 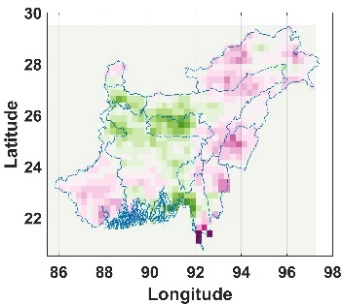 | | | SSP126 | 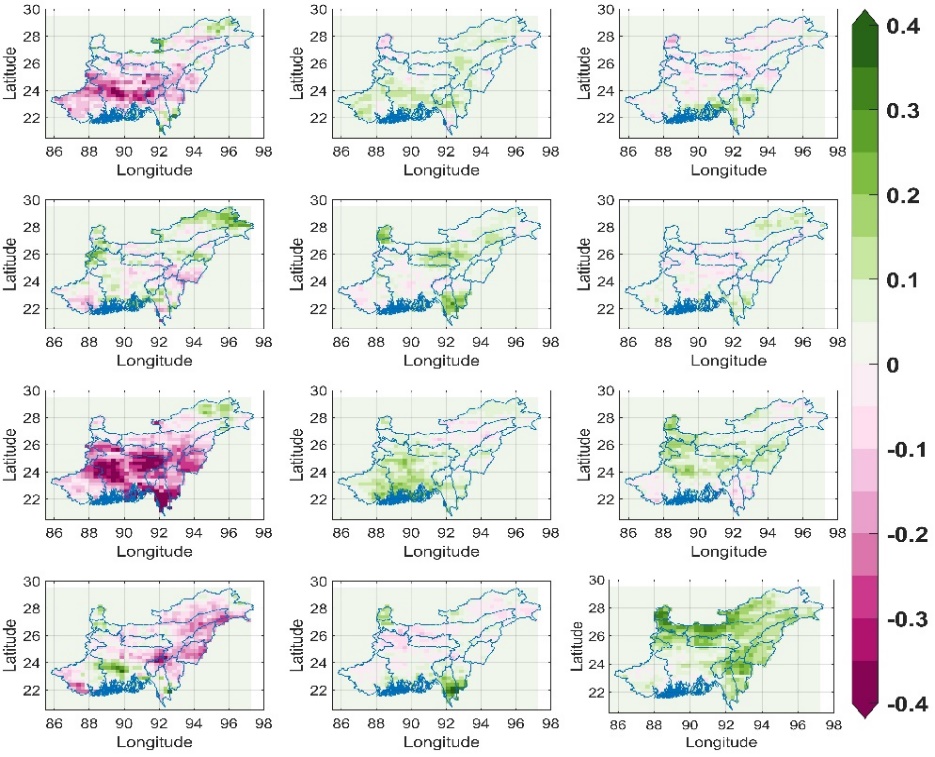 | | | | |
|  |  |  | SSP245 |  |  |  |  |  |
|  |  |  | SSP370 |  |  |  |  |  |
|  |  |  | SSP585 |  |  |  |  |  |

**Figure S13:** **(a)** Change of the CWD (in Days) during three different future Epochs as compared to the reference period, derived from the MME of the 14 GCM models for four SSP scenarios (CMIP6). **(b)** Trend (in Days/year) of CWD during the reference period and three different future Epochs. The green, purple, and white color represent a statistically significant increasing, decreasing trend, and insignificant trend, respectively. The maximum change of CWD in the future is increased (up to 15 days), with a positive trend of up to 0.3 days/year for Epoch 3 under scenario SSP585.

| **a)** | Reference Period  (1981-2014) | | Scenarios | Future Period | | | | |
| --- | --- | --- | --- | --- | --- | --- | --- | --- |
|  |  |  |  | Epoch 1 | Epoch 2 | | | Epoch 3 |
| **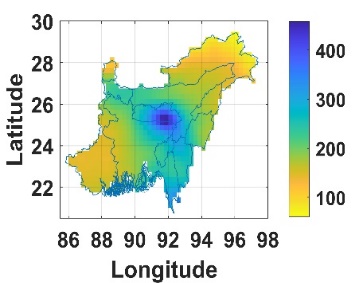** | | | SSP126 | 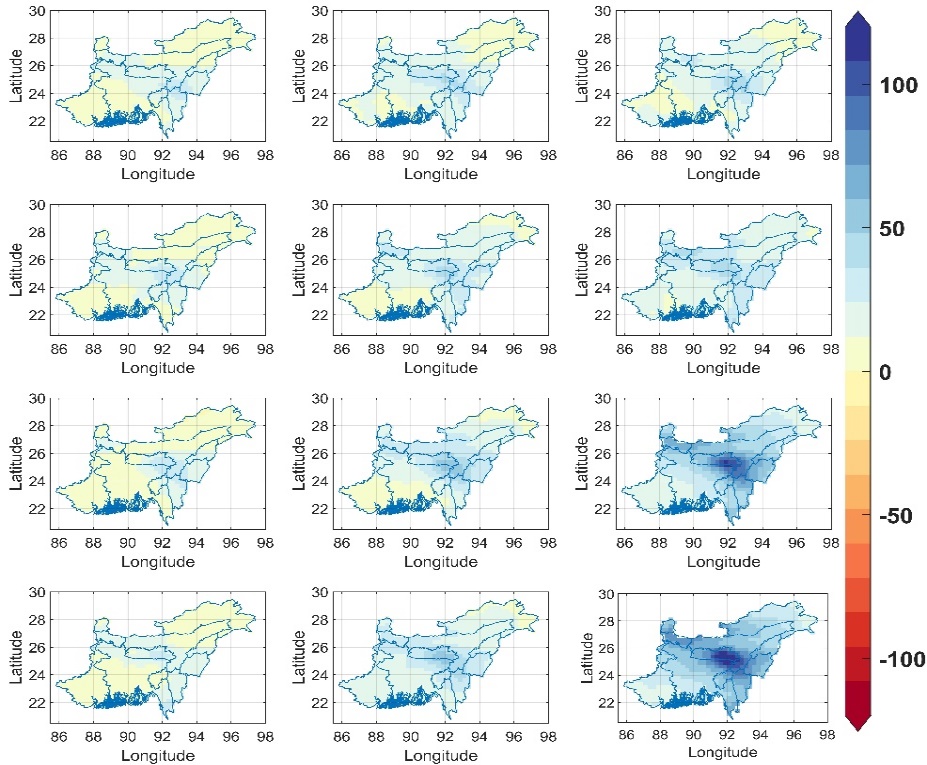 | | | | |
|  |  |  | SSP245 |  |  |  |  |  |
|  |  |  | SSP370 |  |  |  |  |  |
|  |  |  | SSP585 |  |  |  |  |  |
| **b)** | | Reference Period | | Epoch 1 | | Epoch 2 | Epoch 3 | |
| 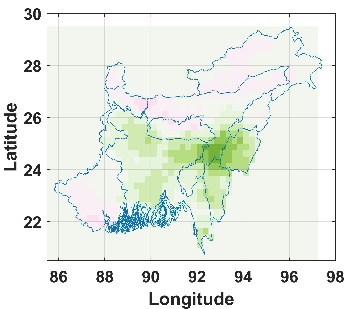 | | | SSP126 | 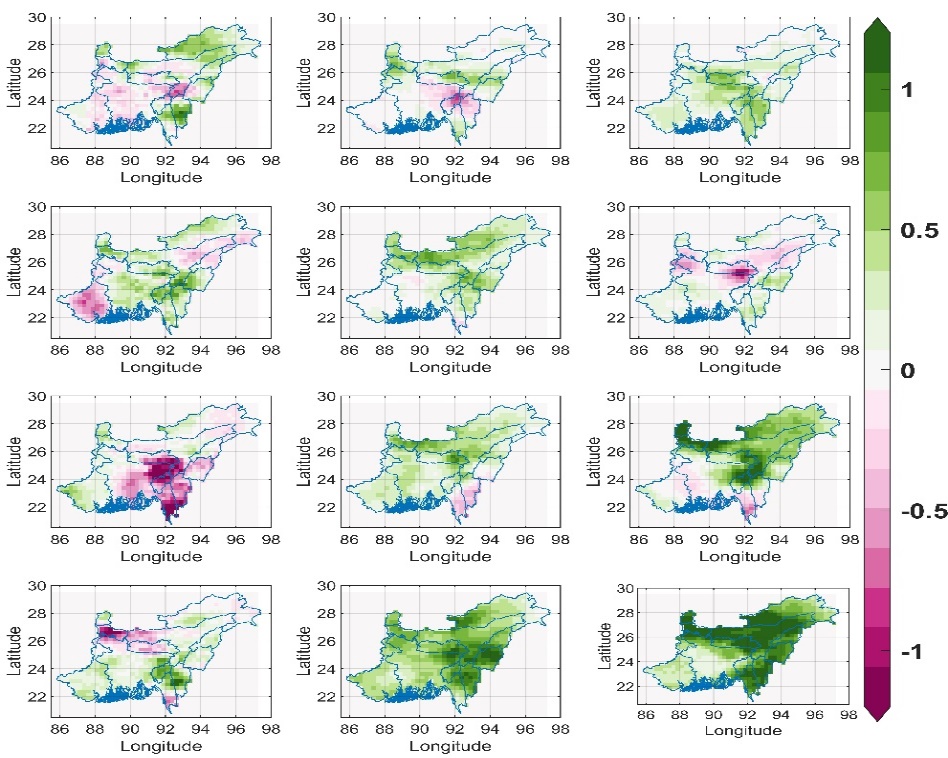 | | | | |
|  |  |  | SSP245 |  |  |  |  |  |
|  |  |  | SSP370 |  |  |  |  |  |
|  |  |  | SSP585 |  |  |  |  |  |

**Figure S14:** **(a)** Change of the Rx5day (in mm) during three different future Epochs as compared to the reference period, derived from the MME of the 14 GCM models for four SSP scenarios (CMIP6). **(b)** Trend (in mm/year) of Rx5day during the reference period and three future Epochs. The green, purple, and white color represent a statistically significant increasing, decreasing trend, and insignificant trend, respectively. The maximum change of Rx5day in the future is increased (up to 120 mm), with a positive trend of up to 1.6 mm/year for Epoch 3 under scenario SSP585.

| **a)** | Reference Period  (1981-2014) | | Scenarios | Future Period | | | | |
| --- | --- | --- | --- | --- | --- | --- | --- | --- |
|  |  |  |  | Epoch 1 | Epoch 2 | | | Epoch 3 |
| **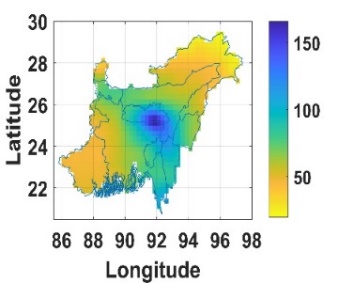** | | | SSP126 | 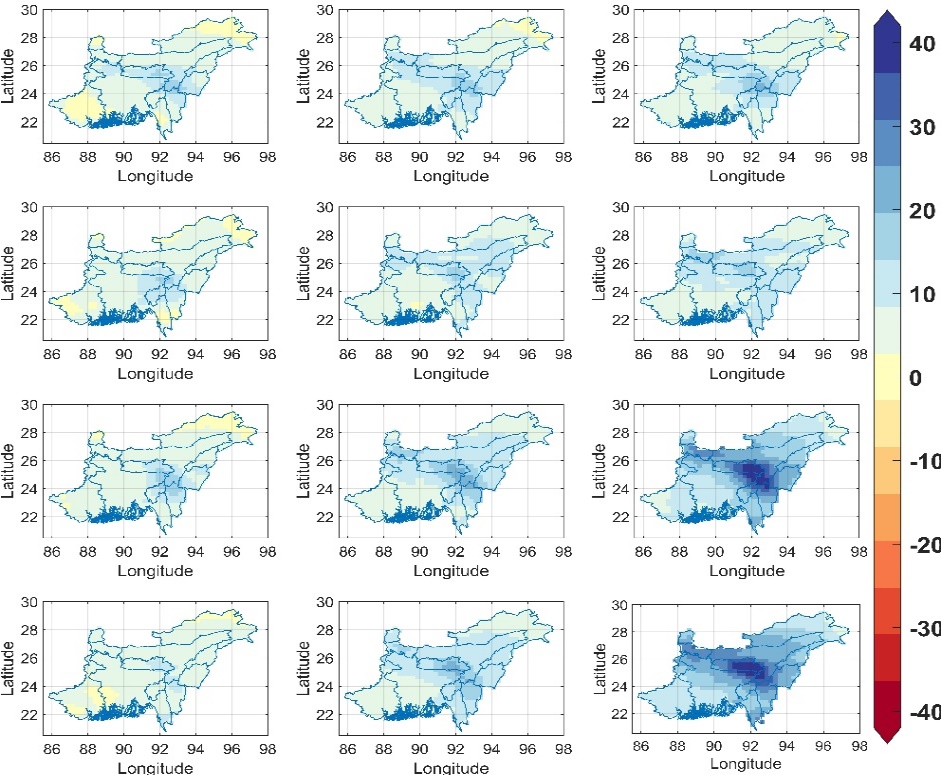 | | | | |
|  |  |  | SSP245 |  |  |  |  |  |
|  |  |  | SSP370 |  |  |  |  |  |
|  |  |  | SSP585 |  |  |  |  |  |
| **b)** | | Reference Period | | Epoch 1 | | Epoch 2 | Epoch 3 | |
| 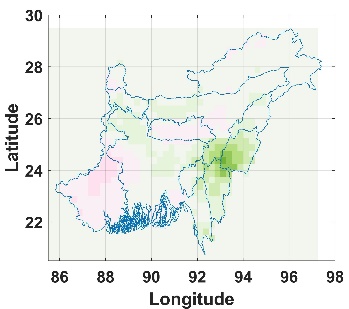 | | | SSP126 | 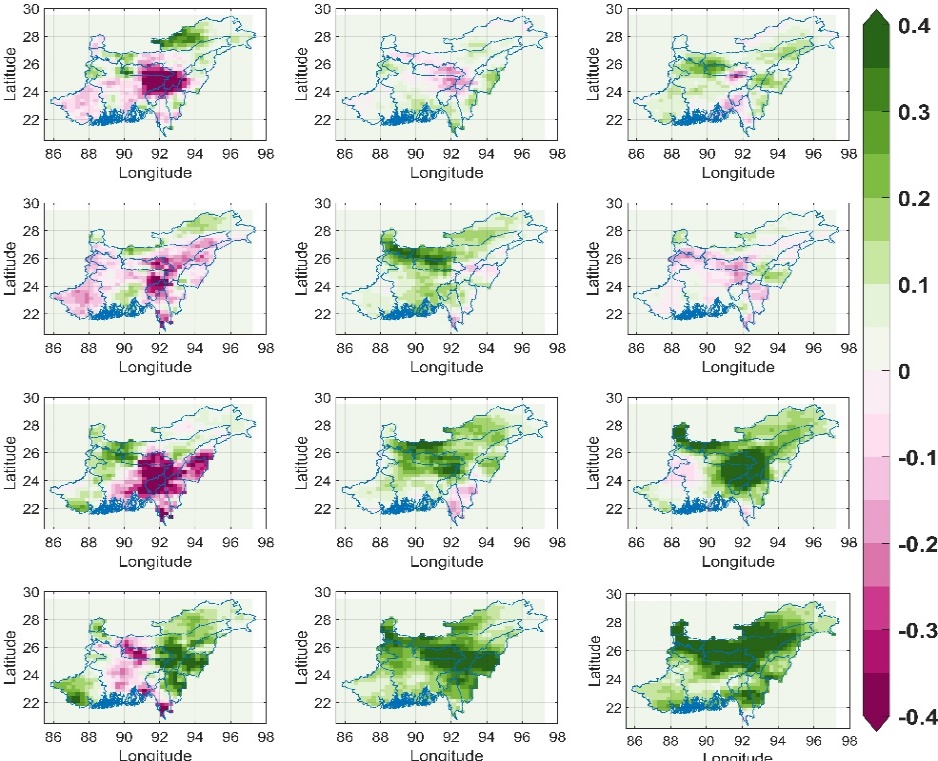 | | | | |
|  |  |  | SSP245 |  |  |  |  |  |
|  |  |  | SSP370 |  |  |  |  |  |
|  |  |  | SSP585 |  |  |  |  |  |

**Figure S15:** **(a)** Change of the Rx1day (in mm) during three different future Epochs as compared to the reference period, derived from the MME of the 14 GCM models for four SSP scenarios (CMIP6). **(b)** Trend (in mm/year) of Rx1day during the reference period and three future Epochs. The green, purple, and white colors represent a statistically significant increasing, decreasing, and insignificant trend, respectively. The maximum change of Rx1day in the future is increased (up to 42mm), with a positive trend of up to 0.6 mm/year for Epoch 3 under scenario SSP585.

| **a)** | Reference Period  (1981-2014) | | Scenarios | Future Period | | | | |
| --- | --- | --- | --- | --- | --- | --- | --- | --- |
|  |  |  |  | Epoch 1 | Epoch 2 | | | Epoch 3 |
| **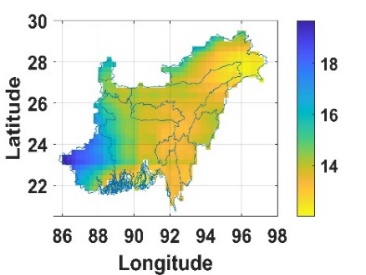** | | | SSP126 | 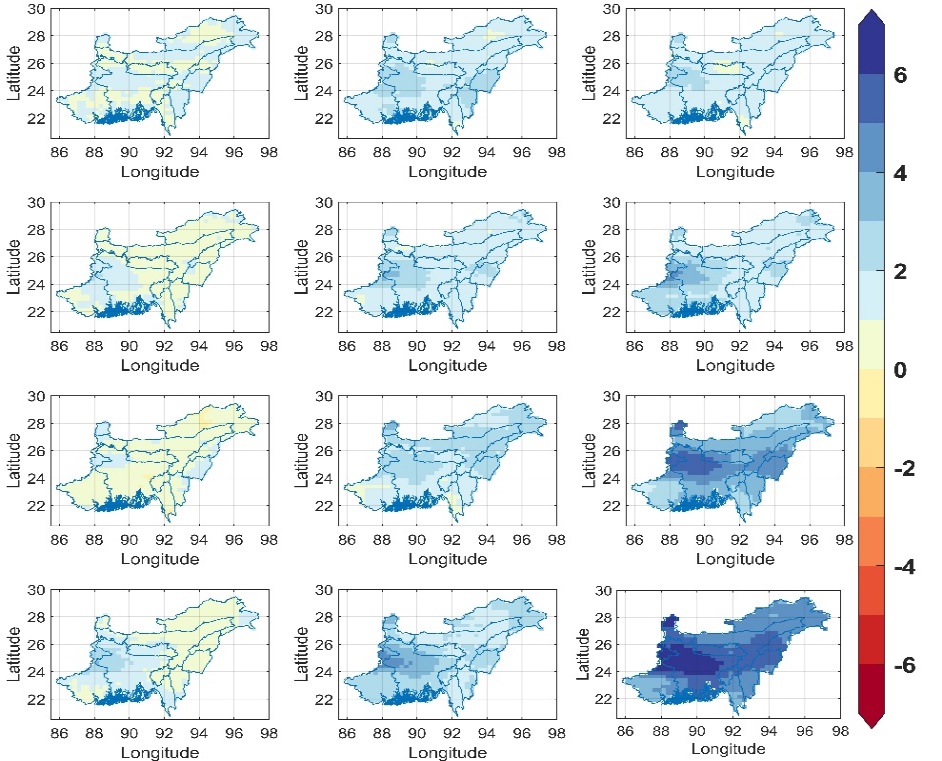 | | | | |
|  |  |  | SSP245 |  |  |  |  |  |
|  |  |  | SSP370 |  |  |  |  |  |
|  |  |  | SSP585 |  |  |  |  |  |
| **b)** | | Reference Period | | Epoch 1 | | Epoch 2 | Epoch 3 | |
| 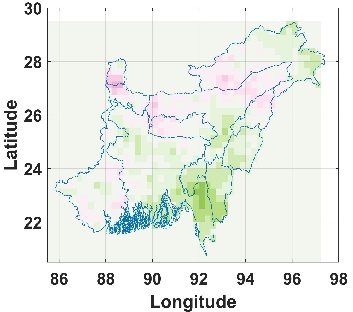 | | | SSP126 | 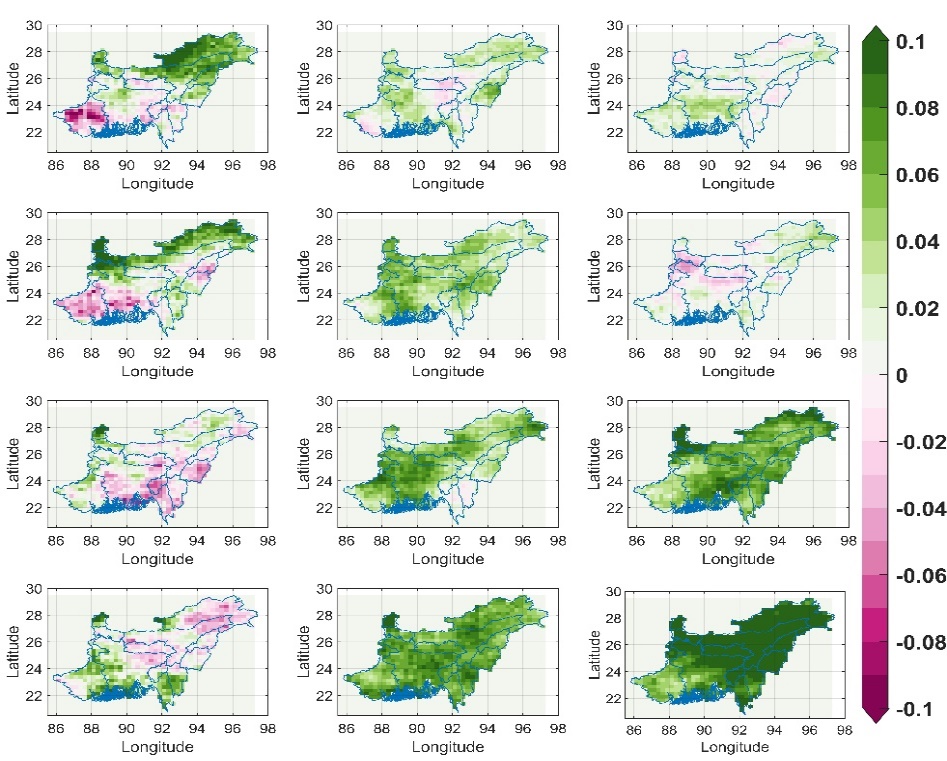 | | | | |
|  |  |  | SSP245 |  |  |  |  |  |
|  |  |  | SSP370 |  |  |  |  |  |
|  |  |  | SSP585 |  |  |  |  |  |

**Figure S16: (a)** Change of the R95P (in %) during three different future Epochs as compared to the reference period, derived from the MME of the 14 GCM models for four SSP scenarios (CMIP6). **(b)** Trend (in %/year) of R95P during the reference period and three future Epochs. The green, purple, and white color represent a statistically significant increasing, decreasing trend, and insignificant trend, respectively. The maximum change of R95p in the future is increased (up to 8%), with a positive trend of up to 0.085%/year for Epoch 3 under scenario SSP585.

| **a)** | Reference Period  (1981-2014) | | Scenarios | Future Period | | | | |
| --- | --- | --- | --- | --- | --- | --- | --- | --- |
|  |  |  |  | Epoch 1 | Epoch 2 | | | Epoch 3 |
| **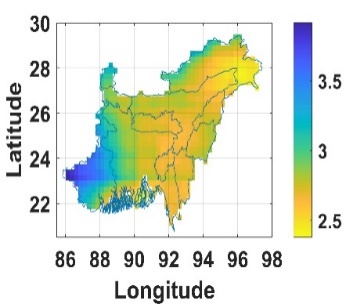** | | | SSP126 | 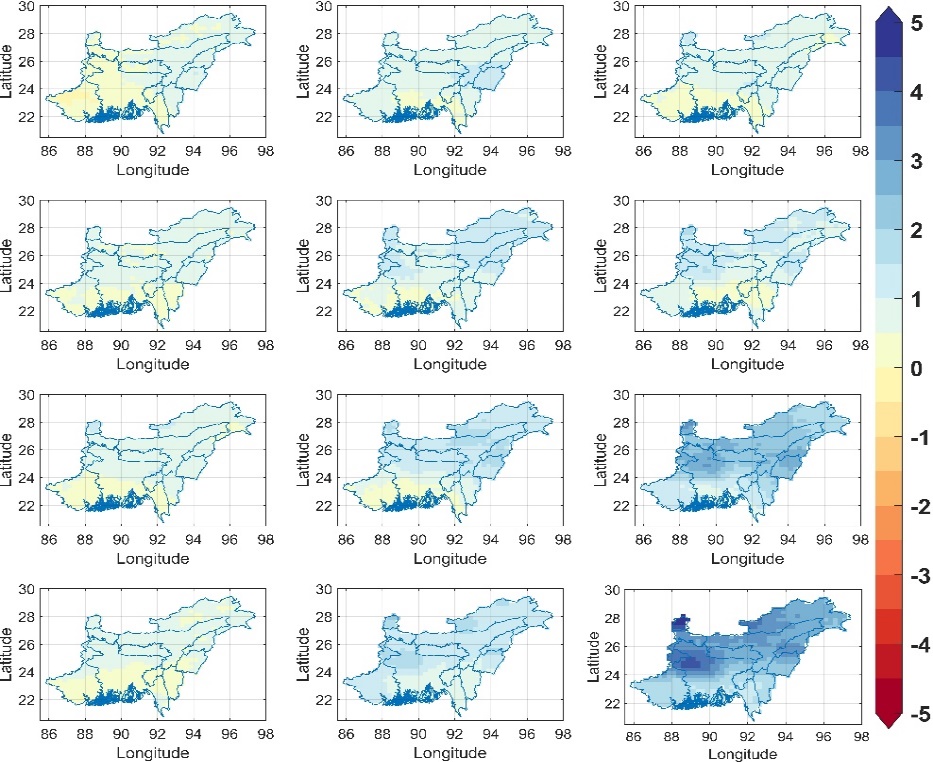 | | | | |
|  |  |  | SSP245 |  |  |  |  |  |
|  |  |  | SSP370 |  |  |  |  |  |
|  |  |  | SSP585 |  |  |  |  |  |
| **b)** | | Reference Period | | Epoch 1 | | Epoch 2 | Epoch 3 | |
| 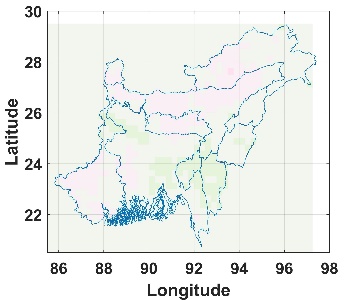 | | | SSP126 | 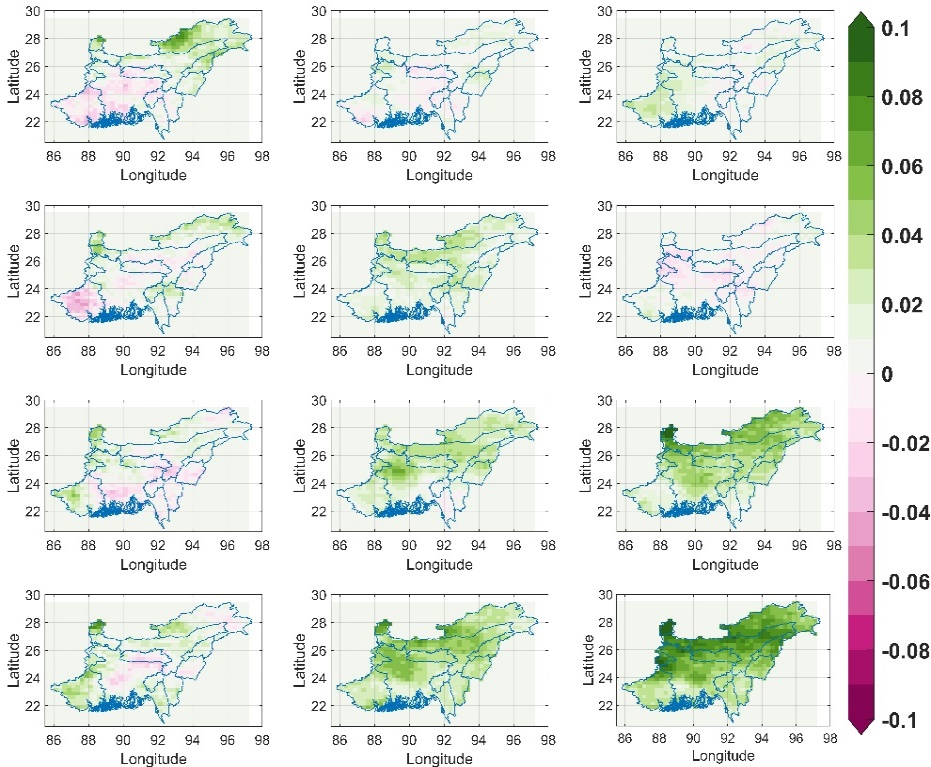 | | | | |
|  |  |  | SSP245 |  |  |  |  |  |
|  |  |  | SSP370 |  |  |  |  |  |
|  |  |  | SSP585 |  |  |  |  |  |

**Figure S17:** **(a)** Change of the R99P (in %) during three different future Epochs as compared to the reference period, derived from the MME of the 14 GCM models for four SSP scenarios (CMIP6). **(b)** Trend (in %/year) of R99P during the reference period and three future Epochs. The green, purple, and white color represent a statistically significant increasing, decreasing trend, and insignificant trend, respectively. The maximum change of R99p in the future is increased (up to 5%), with a positive trend of up to 0.065%/year for Epoch 3 under scenario SSP585.

| **a)** | Reference Period  (1981-2014) | | Scenarios | Future Period | | | | |
| --- | --- | --- | --- | --- | --- | --- | --- | --- |
|  |  |  |  | Epoch 1 | Epoch 2 | | | Epoch 3 |
| **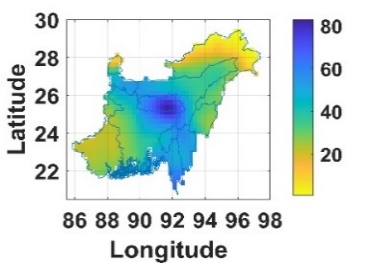** | | | SSP126 | 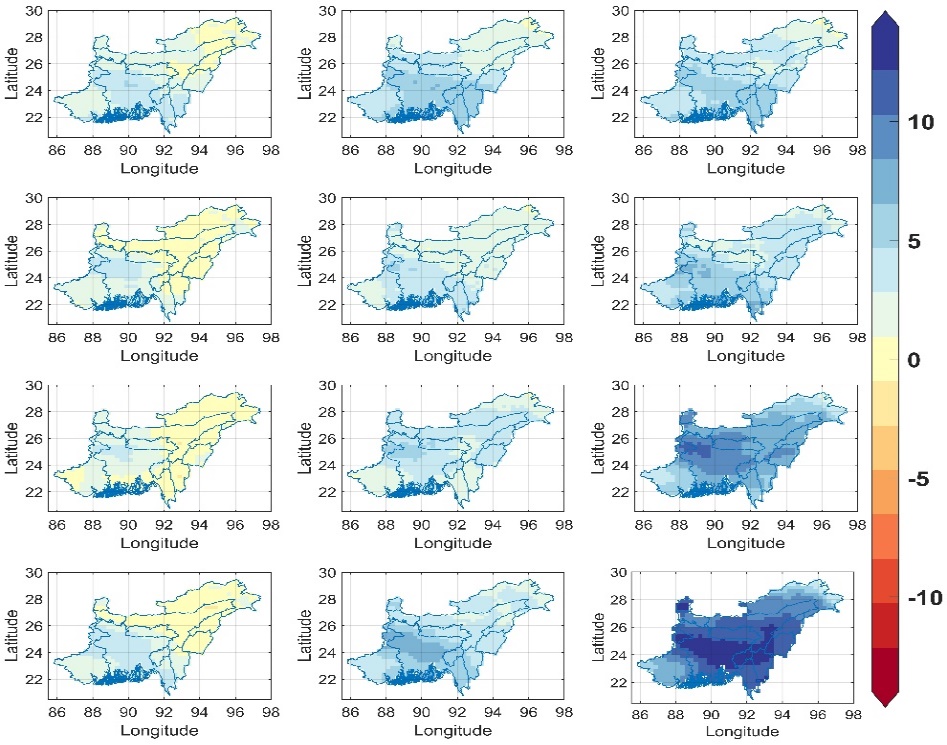 | | | | |
|  |  |  | SSP245 |  |  |  |  |  |
|  |  |  | SSP370 |  |  |  |  |  |
|  |  |  | SSP585 |  |  |  |  |  |
| **b)** | | Reference Period | | Epoch 1 | | Epoch 2 | Epoch 3 | |
| 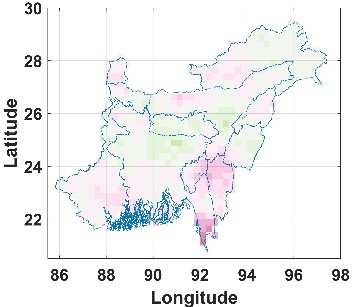 | | | SSP126 | 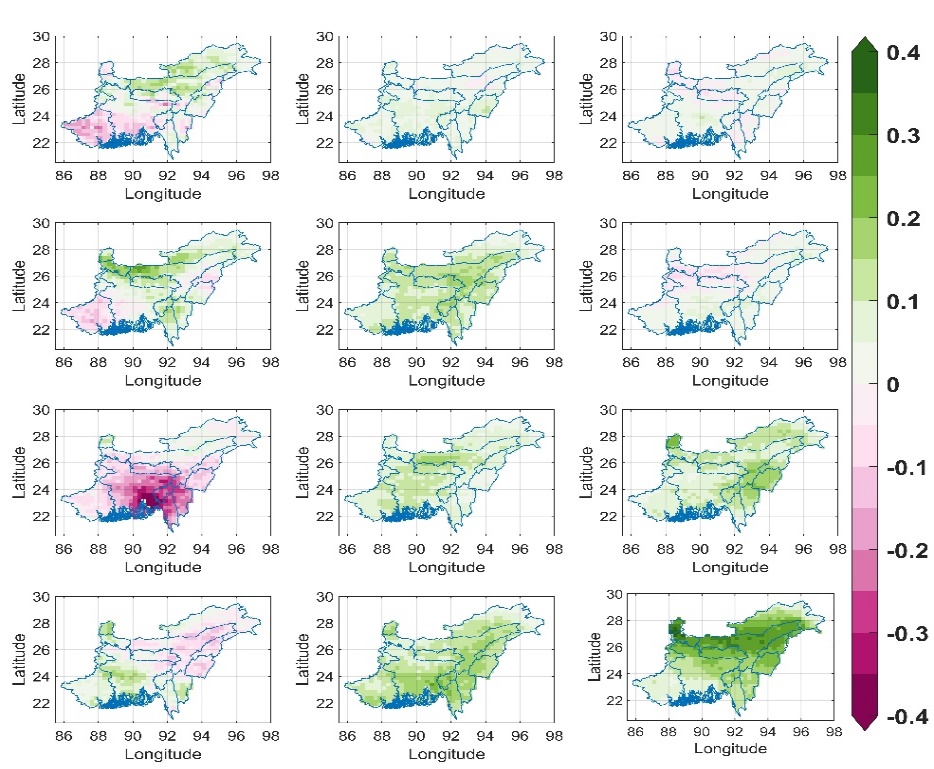 | | | | |
|  |  |  | SSP245 |  |  |  |  |  |
|  |  |  | SSP370 |  |  |  |  |  |
|  |  |  | SSP585 |  |  |  |  |  |

**Figure S18:** **(a)** Change of the R20mm (in Days) during three different future Epochs as compared to the reference period, derived from the MME of the 14 GCM models for four SSP scenarios (CMIP6). **(b)** Trend (in Days/year) of R20mm during the reference period and three future Epochs. The green, purple, and white color represent a statistically significant increasing, decreasing trend, and insignificant trend, respectively. The maximum change of R20mm in the future is increased (up to 14 days) with a positive trend of up to 0.3 days/year for Epoch 3 under scenario SSP585.

1. Corresponding author: Rajib Maity, email [rajib@civil.iitkgp.ac.in](mailto:rajib@civil.iitkgp.ac.in), ORCID: 0000-0001-5631-9553 [↑](#footnote-ref-1)
